# Inside-out assembly of viral antigens for the enhanced vaccination

**DOI:** 10.1038/s41392-023-01414-7

**Published:** 2023-05-24

**Authors:** Fengqiang Cao, Sha Peng, Yaling An, Kun Xu, Tianyi Zheng, Lianpan Dai, Kenji Ogino, To Ngai, Yufei Xia, Guanghui Ma

**Affiliations:** 1 State Key Laboratory of Biochemical Engineering, Institute of Process Engineering, Chinese Academy of Sciences, Beijing, 100190 PR China; 2 Graduate School of Bio-Applications and Systems Engineering, Tokyo University of Agriculture and Technology, 2-24-16 Nakacho, Koganei, Tokyo, 184-8588 Japan; 3 Institute of Global Innovation Research, Tokyo University of Agriculture and Technology, 2-24-16 Nakacho, Koganei-shi, Tokyo, 184-8588 Japan; 4 CAS Key Laboratory of Pathogenic Microbiology and Immunology, Institute of Microbiology, Chinese Academy of Sciences, Beijing, 100101 PR China; 5 Savaid Medical School, University of Chinese Academy of Sciences, Beijing, PR China; 6 Key Laboratory of Tropical Translational Medicine of Ministry of Education, School of Tropical Medicine and Laboratory Medicine, The First Affiliated Hospital, Hainan Medical University, Hainan, 571199 PR China; 7 Zhejiang University School of Medicine, Hangzhou, 310058 PR China; 8 Department of Chemistry, The Chinese University of Hong Kong, Shatin, N. T. Hong Kong, 999077 PR China; 9 University of Chinese Academy of Sciences, Beijing, 100049 PR China; 10 Innovation Academy for Green Manufacture Chinese Academy of Sciences, Beijing, 100190 PR China

**Keywords:** Vaccines, Drug delivery

## Abstract

Current attempts in vaccine delivery systems concentrate on replicating the natural dissemination of live pathogens, but neglect that pathogens evolve to evade the immune system rather than to provoke it. In the case of enveloped RNA viruses, it is the natural dissemination of nucleocapsid protein (NP, core antigen) and surface antigen that delays NP exposure to immune surveillance. Here, we report a multi-layered aluminum hydroxide-stabilized emulsion (MASE) to dictate the delivery sequence of the antigens. In this manner, the receptor-binding domain (RBD, surface antigen) of the spike protein was trapped inside the nanocavity, while NP was absorbed on the outside of the droplets, enabling the burst release of NP before RBD. Compared with the natural packaging strategy, the inside-out strategy induced potent type I interferon-mediated innate immune responses and triggered an immune-potentiated environment in advance, which subsequently boosted CD40^+^ DC activations and the engagement of the lymph nodes. In both H1N1 influenza and SARS-CoV-2 vaccines, rMASE significantly increased antigen-specific antibody secretion, memory T cell engagement, and Th1-biased immune response, which diminished viral loads after lethal challenge. By simply reversing the delivery sequence of the surface antigen and core antigen, the inside-out strategy may offer major implications for enhanced vaccinations against the enveloped RNA virus.

## Introduction

A major challenge in vaccine design is stimulating the potency and duration of the immune responses.^1,2^ The immune responses to infection or vaccination are temporal sequences of events, which depend on the ordered exposure of antigenic components to the immune system,^3,4^ as well as the coordinated actions of the lymph nodes, immunocytes, cytokines, etc.^5,6^ Thus, potent vaccines are expected to harness spatial and temporal control over sequential immune activation.^7^

To address this, nano- and micro-delivery systems with controllable physicochemical properties and multi-level nanostructures are engineered to deliver multiple vaccine components.^8,9^ Additionally, since pathogens are the perfect vehicles of natural selection, there is a trend to mimic their structures or physiochemical properties.^10,11^ Increased lymph node accumulation of antigen, antigen uptake, and antigen cross-presentation have been witnessed in previous attempts to replicate live pathogens’ sizes, shapes, charges, and softness.^12,13^ As for the delivery kinetics, it is thought to replicate the natural dissemination of multiple antigenic components, which may dictate the exposure sequence for subsequent immune activation in a biomimetic manner.^14^

Nonetheless, pathogens usually evolve to escape the immune system rather than to provoke it.^15–17^ In the case of enveloped RNA viruses, genome replication results in the accumulation of pathogen-associated molecular patterns, which can lead to a strong host anti-viral response.^18,19^ To circumvent this, immunogenic components, such as viral genes and proteins (e.g., nucleocapsid protein, NP), are tightly bound and hidden inside.^20,21^ Subsequently, the embedded NP is delayed in its exposure to immune surveillance, leading to suppressed type I interferon (IFN-I) expression, as well as impeded anti-viral effects.^22^ Accordingly, the exact replicas of natural dissemination may not be an optimal solution. As a preliminary test, we treated bone marrow-derived dendritic cells (BMDCs) with the surface antigen and NP of H1N1 influenza virus (A/Puerto Rico/8/1934)^23^ and SARS-CoV-2 (hCoV-19/China/CAS-B001/2020),^24^ respectively. In the presence of the surface antigen, higher doses of NP resulted in the up-regulated expression of IFN-α, suggesting a robust anti-viral effect (Supplementary Fig. 1).

Under these circumstances, we anticipated that it would be the natural packaging of NPs on the inside and surface antigens on the outside, which delays the exposure of NPs to immune surveillance. Instead, the inside-out assembly of the viral antigens, which enables the exposure of the core antigens before the surface antigens (reversed delivery), may potentiate the immune responses. Compared to the exact replicas of the natural dissemination, the inside-out strategy may trigger a more robust IFN-I-mediated innate immune response in advance, cultivating an immune stimulatory environment for enhanced potency and duration of the immune responses. To this end, the delivery system is expected to offer a multi-level landing spot for the ordered and inside-out assembly of viral antigens with high loading efficiency, which may offer a tunable release at the specific location and the right time, thus dictating IFN-I signaling. Moreover, to maintain the protein structure and immunogenicity, it is also imperative to provide a facile and mild loading method to avoid the involvement of high-shear stress or organic solvents.

**Fig. 1:**
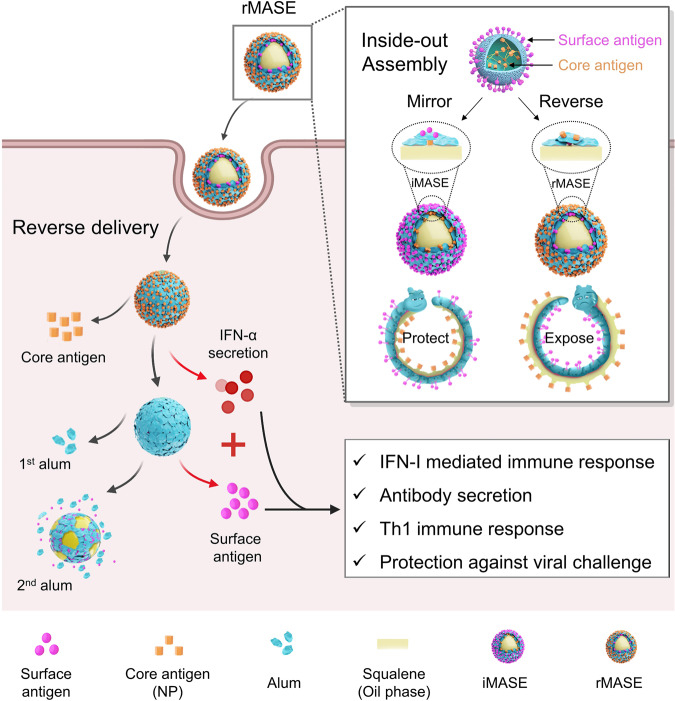
Schematic illustration of the inside-out assembly of the viral antigens. Compared with the natural dissemination (iMASE, left), the reversed delivery (rMASE, right) allowed for the prior encountering of NP to the immune surveillance, as hidden caterpillars (pathogens) were forced to expose their “soft spot” to the predators (immune systems), provoking IFN-I secretion in advance. Along with the delivered surface antigen, rMASE promoted the innate immune response for enhanced antibody secretion and antigen-specific T cell immune response against viral challenge

To achieve this, we developed a multi-layered alum-stabilized emulsion (MASE) to harness the delivery kinetics of the surface and core antigens. Through the co-assembly of alum and antigen at the oil/water (o/w) interface, the core antigen was trapped within the nanocage formed by the alum and o/w interface. Subsequently, another layer of alum was deposited, which further shielded the inner antigen and provided adsorption sites for the outer antigen. As such, the embedded antigen was only released after the detachment of the deposited alum, thus constituting the sequential delivery system. On the o/w interface, the layer-by-layer assembly may bypass the multiple encapsulation procedures and the involvement of organic reagents, assuring the epitope integrity of the proteins and the consecutive loading of surface antigen and NP in a facile and moderate way. To demonstrate the natural dissemination, surface antigen and NP were assembled consecutively on the outside and inside of the multi-layered droplets (iMASE). In contrast, the inside-out assembly reversed the delivery of surface antigen and NP (rMASE), thereby exposing the “soft spot” of the viruses (depicted as the caterpillar in Fig. 1). Consequently, it is anticipated that the inside-out strategy can cultivate the reversed encounter of the surface and core antigens to the immune system, which may strongly stimulate the anti-viral host immune responses. In this manner, IFN-I-mediated innate immune response may be activated for enhanced adaptive immune responses.

## Results

### Tailoring multi-layer alum-stabilized emulsions for the inside-out assembly of the antigens

**Fig. 2:**
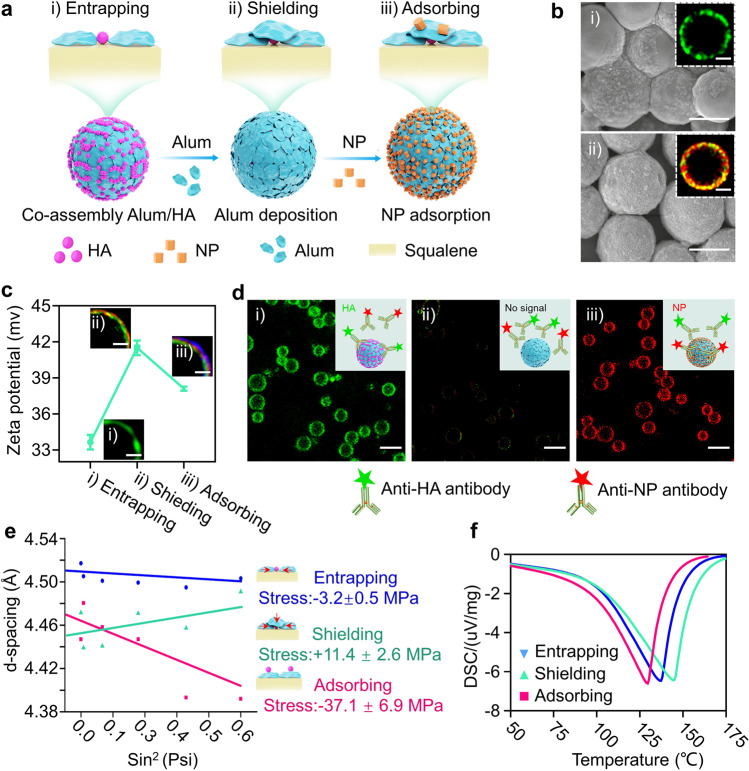
Tailoring rMASE for the inside-out strategy. **a** Schematic illustration on rMASE preparation for the inside-out assembly of HA and NP. Stepwise formation of rMASE. (i) Co-assembly of alum and HA on the o/w interface; (ii) another layer of alum deposition to shield the inner HA; (iii) NP adsorption to constitute the sequential loading of HA and NP to finally obtain rMASE. **b** SEM (scale bar: 2 µm) and STED (scale bar: 1 µm) images of co-assembly alum/HA (i) and alum deposition (ii). **c** Zeta potential and structure illumination microscopy (SIM) images of rMASE. HA, NP, and alum were labeled with Cy3 (green), Cy5 (blue), and lumogallion (red), respectively. Scale bar: 1 µm. Data were shown as mean ± s.e.m (*n* = 3, from 3 independent experiments). **d** Verifying the thorough coverage of the inner HA and the surface display of NP for the inside-out strategy. The droplets were treated with 4% (v/v) FBS solution to avoid non-specific interaction and then with a mixture of anti-HA antibody (green) and anti-NP antibody (red), followed by confocal imaging. Scale bar: 5 µm. **e** XRD analysis on surface residual stress of droplets. The presence of residual stress (*σ*_*φ*_) reflected the force tendency of the inner and outer antigens. The data were analyzed by regressing each data point to a straight line, and the linear slope M was obtained. Measuring and calculating the modulus of elasticity and Poisson’s ratio to calculate K, and the stress can be calculated from *σ*_*φ*_ = *KM*. Residual stress (*σ*_*φ*_) indicated the tendency of the antigen towards (*σ*_*φ*_ > 0, compressive stress) or backwards (*σ*_*φ*_ < 0, tensile stress) the o/w interface. **f** DSC studies on the varied cooperated states of the inner HA. The right-shifting of the thermal peak indicated a greater energy for antigen to escape from the droplets, implying a more impeded release tendency

Here, the inside-out strategy was first tested to deliver hemagglutinin (HA) and NP of the H1N1 influenza virus (A/Puerto Rico/8/1934, Supplementary Table 1). rMASE was prepared according to the schematic illustration (Fig. 2a). By adjusting the pH and type of the continuous phase, alum and HA were co-assembled on the o/w interface, forming alum/HA-assembled droplets (Supplementary Fig. 2). Circular dichroism (CD) analysis revealed that the secondary structure of HA remained unchanged after co-assembly with alum (Supplementary Fig. 3a). Subsequently, as evidenced by the quartz crystal micro-balance with dissipation monitoring (QCM-D), another layer of alum was attached via the interaction with the alum/HA-assembled droplets (Supplementary Fig. 3b). After optimization of the outer layer alum concentration, multi-layered droplets were prepared with no excess alum in the continuous phase, but with enough alum to cover the inner antigen (Supplementary Fig. 4a–c). Unlike the alum/HA-stabilized droplets, scanning electron microscopy (SEM) images demonstrated an increased padding morphology, and stimulated emission depletion microscopy (STED) indicated an additional alum layer (red) on the alum/HA-stabilized droplets, thereby entrapping the inner HA (green) within the layer-by-layer nanostructures (Fig. 2b). In addition, a large surface area was exposed for NP adsorption. Based on the changes in the zeta potentials and elemental compositions, rMASE was prepared with HA and NP loaded inside and outside, respectively (Fig. 2c and Supplementary Fig. 4d, e).

To test whether the inner antigen was completely shielded, the droplets were treated with a mixture of monoclonal antibodies against HA and NP. The images illustrated that alum/HA-stabilized droplets were strongly bonded with anti-HA antibodies (green). Subsequently, the addition of outer layer alum showed an evident reduction in the fluorescent intensity, suggesting the covering of the inner HA to avoid pre-exposure during antigen delivery. Additionally, after NP adsorption, the strong signal of a single fluorescence (red) indicated the dense display of NP on the rMASE surface (Fig. 2d). In a similar fashion, iMASE was prepared to load NP on the inside and adsorb HA on the outside of the droplets, which was determined with similar size and antigen loading efficiency but reversed antigen distribution compared to rMASE (Supplementary Fig. 5). Accordingly, the multi-layered alum-stabilized emulsion was developed for the inside-out assembly of NP and surface antigens. Through the layer-by-layer procedure on the o/w interface, iMASE and rMASE achieved consecutive loading of HA and NP in a facile and moderate manner, demonstrating the natural and reversed antigen distributions of the H1N1 influenza virus (Supplementary Table 2).

### Dictating the release tendency of the antigens

Next, we investigated whether consecutive loading could affect the release kinetics of the outer and inner antigens. To verify the release tendency, the residual stress was evaluated via X-ray diffraction (XRD) analysis (Fig. 2e). Compared to their antigen-adsorbed counterparts (pink line), the co-assembled antigen and alum demonstrated decreased tensile stress. Whereas the attachment of the outer alum layer changed the residual stress from tensile stress to compressive stress, suggesting that the inner antigen was more likely to be entrapped within the nanocage formed between the close-binding alum and the o/w interface. Moreover, the right shift of the thermal peak in differential scanning calorimetry (DSC) demonstrated that a high thermal energy was required for the antigen to escape from the alum/HA-assembled droplets. These results indicated that the inner antigen had an impeded release tendency (Fig. 2f).

Then, rMASE was co-incubated with a 10% (v/v) fetal bovine serum (FBS) solution to test the release profile after administration. To better simulate the interstitial fluid, macromolecules larger than 30 kDa were removed using a centrifugal concentrator (30 kDa MWCO).^25,26^ As shown in Supplementary Fig. 6a, b, a limited amount of antigen was discharged from the droplets, suggesting that the antigens were only released after cellular internalization. With the macromolecules in the system, the outer NP was released before the entrapped HA (Supplementary Fig. 6c–e). Moreover, the release rate increased with increasing FBS concentrations, suggesting that the antigen may be emitted by ligand exchange with fluidic macromolecules (Supplementary Fig. 6f, g).

### Reversed delivery of surface and core antigens

**Fig. 3:**
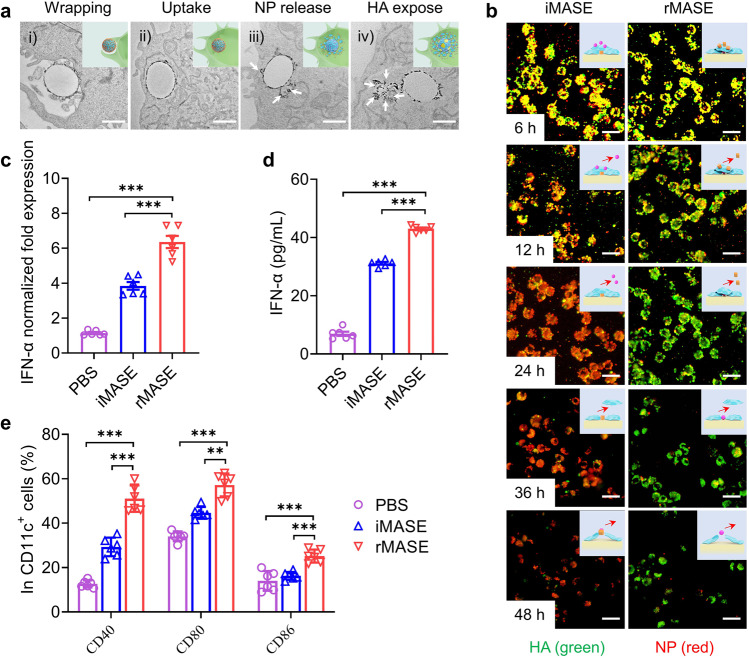
Reversed delivery for the potent IFN-I-mediated immune activation in vitro. **a** Intracellular transfer of rMASE traced by TEM. Arrows indicated the dischargement of the alum. Scale bar: 2 μm. **b** Intracellular release of the antigens monitored via high content screen microscopy. HA and NP were labeled with FITC (green) and Cy5 (red), respectively. Scale bar: 20 µm. The release profile could be assessed by the fluorescence decaying of the loaded antigens. **c** IFN-α mRNA expression levels of the treated DCs at 24 h. **d** IFN-α secretion of the treated DCs at 48 h. **e** The expression of CD40, CD80, and CD86 of the treated DCs at 48 h. All data in the graphs were presented as the arithmetic mean ± s.e.m. from three independent experiments. For statistical analysis, a one-way analysis of variance was conducted with Tukey’s correction for multiple comparisons. *P < 0.05, **P < 0.01, ***P < 0.001

To further assess the release profiles, the intracellular distribution of rMASE was evaluated within BMDCs. As illustrated in the transmission electron microscopy (TEM) images, the droplets were first wrapped by the membrane to increase the contact area, triggering phagocytosis (Fig. 3a, i–ii). With an increase in the specific surface area, the multi-layered droplets stimulated cellular uptake and reached maximum internalization after 6 h (Supplementary Fig. 7a, b). As macromolecular proteins increased within the cytoplasm, the surface alum gradually fell off, along with the release of NP (Fig. 3a, iii). After 24 h, the apparent dissociation of alum occurred, indicating discharge of the inner antigen (Fig. 3a, iv).

Subsequently, the intracellular release of the antigen was monitored using a high content imaging system (Operetta CLS, PerkinElmer). First, HA (FITC-labeled, green) and NP (Cy5-labeled, red) were sequentially loaded. The droplets were subsequently co-incubated with BMDCs for 6 h to achieve the maximum uptake. The fluorescence intensities of the fluidic antigens were too weak to be detected, in contrast to the fluorescence enrichment via micro-sized droplets. Accordingly, the release profile could be assessed by comparing the fluorescence decay of the loaded antigens. In the case of rMASE, a decrease in Cy5 fluorescence intensity demonstrated NP release. After 12 h, the fluorescence intensity of HA started to decline with a primarily constant but gentler slope, representing its subsequent dischargement at a relatively slow rate. Additionally, a reversed trend in iMASE-treated cells suggested the prior release of HA before NP (Fig. 3b and Supplementary Fig. 7c, d). To further verify this, NP-specific immunoglobulin M (IgM) was evaluated. Serum from mice immunized with rMASE exhibited a decreased level of NP-specific IgM on day 14. However, iMASE induced a higher titer on day 14 than on day 7, implying that the release of the inner antigen was delayed for immune recognition (Supplementary Fig. 7e). Thus, by consecutive loading via multi-layered droplets, the inside-out strategy dictated the delivery kinetics of the viral antigens.

To explore the immune effect, the inside-out strategy was tested in BMDCs. Compared with iMASE, rMASE significantly boosted the expression of IFN-α mRNA, with a 138% increase in IFN-α cytokine secretion, indicating the robust activation of IFN-І signaling (Fig. 3c, d). Subsequently, rMASE-treated DCs showed elevated expression of CD40, CD80, and CD86 by 174%, 128%, and 180%, respectively (Fig. 3e). Additionally, both iMASE and rMASE were detected with limited endotoxin levels and cytotoxicity, suggesting that the increased DC activation was attributed to the exposure of NP before HA, instead of potential material contamination or cell damage (Supplementary Fig. 8). Consequently, simply reversing the delivery of HA and NP can promote IFN-I-mediated immune responses.

### Boosting humoral and cellular immune responses against H1N1 influenza

We postulated that rMASE may improve the immune response to H1N1 influenza infection. BALB/c mice were intramuscularly injected once with the formulations indicated in Supplementary Table 3, and the antigen depot was traced over time using an in vivo imaging system. As shown in Supplementary Fig. 9a, an evident antigen depot was observed, comparable with the HA and NP co-adsorbed alum (term “Alum”), and persisted for longer than 3 d. In contrast, the fluidic mixture of HA and NP (term “Antigen”) was cleared from the injection site within 12 h. With the elevated antigen repertoire, DCs were evidently attracted to the injection site for higher antigen uptake (Supplementary Fig. 9b–e). Notably, both iMASE and rMASE demonstrated similar trends in the antigen depot and DC internalization, indicating that the sequential release of viral antigens occurred intracellularly.

**Fig. 4:**
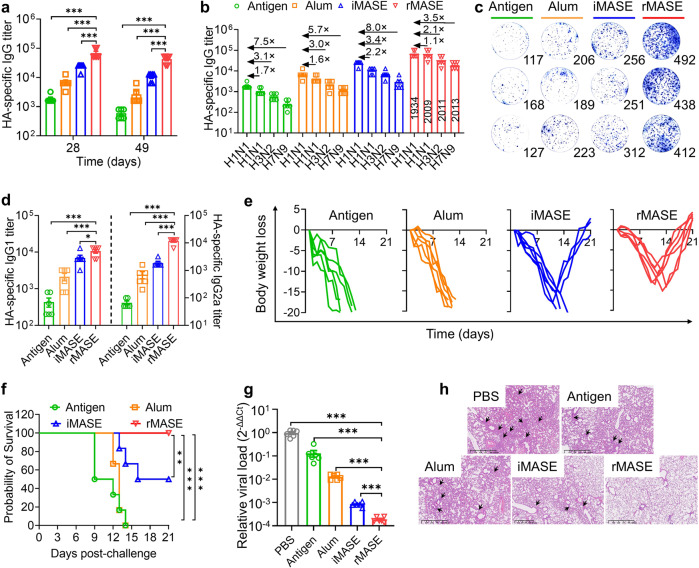
Potent adaptive immune response against H1N1 influenza virus. **a** Serum HA-specific IgG titer. **b** Cross-reactive antibody responses to HA antigens, which derived from A/California/07/2009 (H1N1), A/Hong Kong/3039/2011 (H3N2), and A/Shanghai/ 4664 T/2013 (H7N9), respectively. **c** ELISPOT assay on IFN-γ spot-forming cells among the splenocytes, following stimulation with surface antigen (HA, A/Puerto Rico/8/1934). **d** Serum HA-specific IgG1 and IgG2a levels. **e** Body weight loss after virus challenge. For H1N1 vaccinations, BALB/c mice (*n* = 6) were administrated with the indicated formulations at prime-boost manner (3-week interval) and challenged by influenza virus (A/PR/8/34/1934 (H1N1)) on day 35, 2 times of LD50/mice. **f** Survival rate after viral challenge. Mice that lost ≥20% of their initial body weight were euthanized and counted as dead. **g** Viral loads in the lung by RT-qPCR. **h** Pathological changes in the lung. The sections were stained with H&E. Arrows indicated the perivascular and interstitial infiltration of inflammatory cells and lung consolidation. All data in the graphs were presented as the arithmetic mean ± s.e.m. from three independent experiments. For statistical analysis, a one-way analysis of variance was conducted with Tukey’s correction for multiple comparisons. *P < 0.05, **P < 0.01, ***P < 0.001

After immunizing BALB/c mice intramuscularly twice (three weeks apart), rMASE-induced antigen-specific immune responses were investigated. Notably, rMASE induced significantly higher HA-specific IgG titers compared with Alum (10-fold increase, *P* < 0.001) and iMASE (3-fold increase, *P* < 0.001) after 28 d (Fig. 4a). Additionally, elicitation of a potent serum antibody was also observed after 49 d. To test the cross-reactivity, the serum was also tested on other strains, including A/California/07/2009 (H1N1), A/Hong Kong/3039/2011 (H3N2), and A/Shanghai/4664 T/2013 (H7N9). As shown in Fig. 4b, compared with H1N1 (1934), the antibody titers of iMASE-treated mice showed 220%, 340%, and 800% decreases in H1N1 (2009), H3N2 (2011), and H7N9 (2013) subtypes, respectively. As for rMASE, the reductions were alleviated, with 110%, 210%, and 350% decreases in H1N1 (2009), H3N2 (2011), and H7N9 (2013) subtypes, respectively, suggesting the increased humoral immune responses.

Moreover, reversed delivery of HA and NP promoted cellular immune responses. Compared to iMASE, rMASE increased the presence of IFN-γ-secreting T cells by 162% among splenocytes (*P* < 0.001, Fig. 4c). However, no significant differences were observed in IL-4 secreted cells (Supplementary Fig. 10a). Meanwhile, rMASE elicited a more than 6-fold higher HA-specific IgG2a titer (*P* < 0.001) than iMASE (Fig. 4d). With potent secretion of IL-2, IL-12, and TNF-α, the cytokine profiles further demonstrated that the inside-out strategy stimulated a Th1-biased immune response (Supplementary Fig. 10b). Furthermore, among the rMASE-treated splenocytes, IFN-γ-secreting T cells demonstrated increased cross-reactivity against H1N1 (2009), H3N2 (2011), and H7N9 (2013) subtypes, indicating the enhanced cross-protection against viral mutations (Supplementary Fig. 10c).

To further test immune protection in mice, we challenged the animals with H1N1 strain A/PR/8/34/1934. Weight loss and survival of the animals were monitored for 21 d post-challenge. rMASE-treated mice experienced a slight decrease in mean body weight on day 10, but quickly increased back to normal weight in less than 7 d. All mice in the Antigen- and Alum-treated groups experienced ≥20% weight loss within 13 d. Comparatively, the mean weight loss in the rMASE-treated group was approximately 15.7% of the original weight 10 days post-challenge (Fig. 4e). Notably, the survival rate in the rMASE-treated group was 100%. In contrast, the survival rate was only 50% in the iMASE-treated group, indicating that the reversed delivery of HA and NP increased immune protection against the H1N1 influenza viruses (Fig. 4f). Moreover, reverse transcription quantitative real-time polymerase chain reaction (RT-qPCR) analysis revealed that rMASE-immunized mice had significantly lower amounts of viral RNA in the lung tissues than those immunized with iMASE on day 9 (Fig. 4g). To evaluate pulmonary inflammatory damage, pathological examination was performed using Hematoxylin and Eosin (H&E) staining. As shown in Fig. 4h, no significant infiltration was observed in rMASE-immunized mice. However, iMASE-treated mice developed perivascular and interstitial infiltrates. Next, inflammatory cytokines in the lung were tested. Compared with iMASE, rMASE significantly decreased the levels of MCP-1, IL-8, IL-1β, and IL-6, indicating the alleviation of inflammation (Supplementary Fig. 10d–g). Collectively, the delivery of NP before HA provoked antigen-specific adaptive immune responses against viral infection.

### Robust IFN-I-mediated immune response and lymph node activation

It remains challenging to enhance the long-term immune response and neutralization capabilities against the prevailing mutant strains of SARS-CoV-2. After finding an increase in adaptive immune response and cross-reactivity in H1N1 influenza vaccines, we postulated that the inside-out strategy may also improve the immune potency and duration of SARS-CoV-2 vaccination. Here, the multi-layer alum-stabilized emulsion shielded the surface antigen (RBD) on the o/w interface and adsorbed the core antigen (NP) on the outside (rMASE), allowing for the prior release of NP before RBD (Supplementary Fig. 11a–c and Supplementary Table 4). By successive loading of the viral antigens, iMASE achieved higher release concentrations of RBD before NP (Supplementary Fig. 11d, e).

**Fig. 5:**
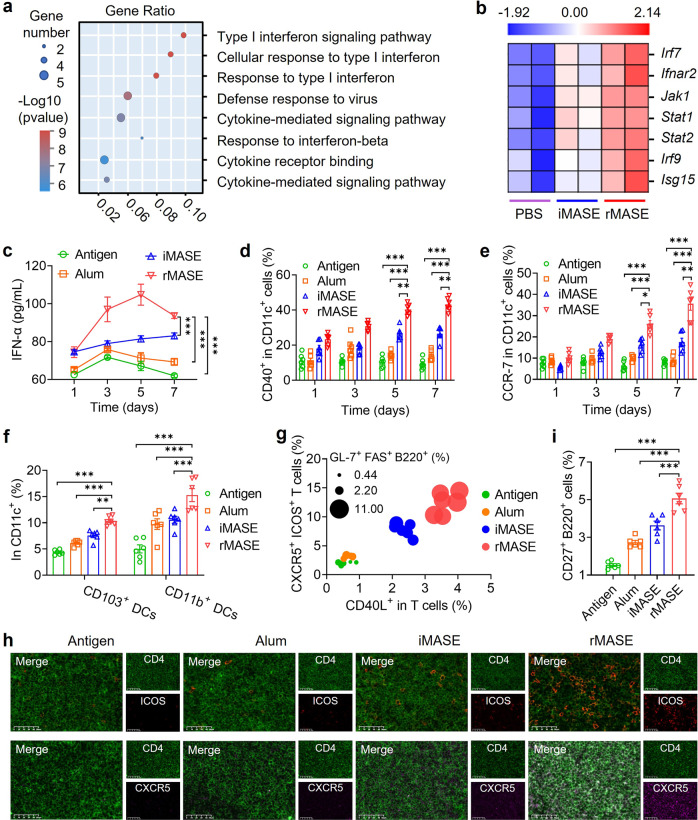
Provoking IFN-I activation in advance for the robust local reaction and lymph node activation. **a** GO term enrichment of the differentially expressed genes between iMASE and rMASE. GO analysis of differentially expressed genes within clusters identified the top associated enriched GO terms with corresponding enrichment *P* values. **b** Transcriptome analysis of DCs after co-culture with iMASE and rMASE. Representative heatmap showed differentially expressed genes relevant to the IFN-I signaling pathway. **c** IFN-α concentrations at the injection site over time. **d** Frequencies of CD40 expressions among the recruited DCs. **e** CCR-7 expressions among the recruited DCs, indicating the LN tropism. **f** The DC subsets within lymph nodes. **g** The bubble plot displays the engagement of the CD40L, germinal center: follicular T helper cells and GC B cells in LN. **h** Representative images of ICOS and CXCR5 immunofluorescence staining in LN. Sections were stained for anti-mouse CD4 antibody (green) and anti-mouse ICOS (red) antibody. The other sections were stained for anti-mouse CD4 antibody (green) and anti-mouse CXCR5 antibody (pink). Scale bar: 50 μm. **i** Memory B cell populations within the LN. All data in the graphs were presented as the arithmetic mean ± s.e.m. from three independent experiments. One-way and two-way analyses of variance were conducted with Tukey’s correction for multiple comparisons. *P < 0.05, **P < 0.01, ***P < 0.001

To test whether the inside-out assembly of RBD and NP also provoked IFN-I-mediated immune responses, their impact on the transcriptome profile was assessed. The gene ontology (GO) term enrichment analysis revealed that rMASE significantly increased the IFN-I-related signaling pathway (under the criteria of *P* ≤ 0.05; Fig. 5a). Furthermore, comparative gene signature analysis revealed that interferon regulatory factor 7 (*Irf7*) was differentially expressed among rMASE-treated DCs, triggering the activation of a series of IFN-stimulated genes (*Jak1*, *Stat1*, *Stat2*, *Irf9*, and *Isg15*; Fig. 5b).

After intramuscular administration (Supplementary Table 5), rMASE significantly boosted the secretion of IFN-α (*P* < 0.001) and IFN-β (*P* < 0.001) at the injection site compared with iMASE (Fig. 5c and Supplementary Fig. 12a). As a consequence of IFN-I-mediated innate immune responses, higher levels of IL-2 (*P* < 0.001, Supplementary Fig. 12b) and TNF-α (*P* < 0.001; Supplementary Fig. 12c) secretions were observed in the early stage after administration, cultivating a more robust immunogenic microenvironment.^27,28^ In response to this, the expression of CD40 and CD86 in the recruited DCs was evidently increased, suggesting enhanced DC activation (Fig. 5d and Supplementary Fig. 12d, e). Notably, no evident inflammation or abnormal levels of IL-6, IL-17A, or MPC-1 were observed, indicating acceptable biosafety and well-controlled immunogenicity (Supplementary Fig. 13). Thus, the inside-out strategy stimulated IFN-I-mediated pathways, which cultivated an immune-stimulatory environment for the onset of an anti-viral state.^29,30^

Then, the activation of the draining lymph nodes (LNs) was probed.^31^ At the injection site, rMASE increased the expression of CCR-7 on the recruited DCs by 195% after 7 d post-administration compared to iMASE, indicating that the recruited DCs have a high potential for migration to LNs (Fig. 5e).^32^ Accordingly, rMASE showed a lower elevated LN-resident DCs proportion (CD8α^+^ CD11c^+^), but a more noticeable increase in the number of migrated DCs (CD103^+^ CD11c^+^ and CD11b^+^ CD11c^+^) within the LNs (Fig. 5f and Supplementary Fig. 14a, b). This indicated that rMASE promoted potent DCs migration from the injection site to the LN to achieve higher LN-accumulation of the antigens, rather than the direct delivery of antigens. Regarding DC activation within the LNs, CD40^+^ DCs were boosted by 150% on the 7th day after the administration of rMASE, compared with the iMASE-treated mice (Supplementary Fig. 14c, d). Furthermore, a significantly higher CD40L expression (*P* < 0.001) among the LN-residing CD3^+^ T cells suggested the increased interactions between DCs and T cells.^33^ Consequently, the rMASE-treated group exhibited a notable expansion of CXCR5^+^ ICOS^+^ CD3^+^ T cells and FAS^+^ GL-7^+^ B220^+^ B cells with an approximate 150% and 200% elevation, respectively (Fig. 5g and Supplementary Fig. 15a–d). Meanwhile, the LN immunofluorescence staining also demonstrated a similar trend, suggesting robust activation of the germinal center (GC; Fig. 5h). Furthermore, we also found that rMASE induced 140% more CD27^+^ B220^+^ cells, compared to iMASE, demonstrating an increase in memory B cells (Fig. 5i and Supplementary Fig. 15e). Thus, the inside-out strategy induced a higher IFN-I-mediated immune response at the early stage of vaccination, which subsequently led to potent LN activation for the onset of a strengthened adaptive immune response.

### Activations on the long-term immune protection against SARS-CoV-2

**Fig. 6:**
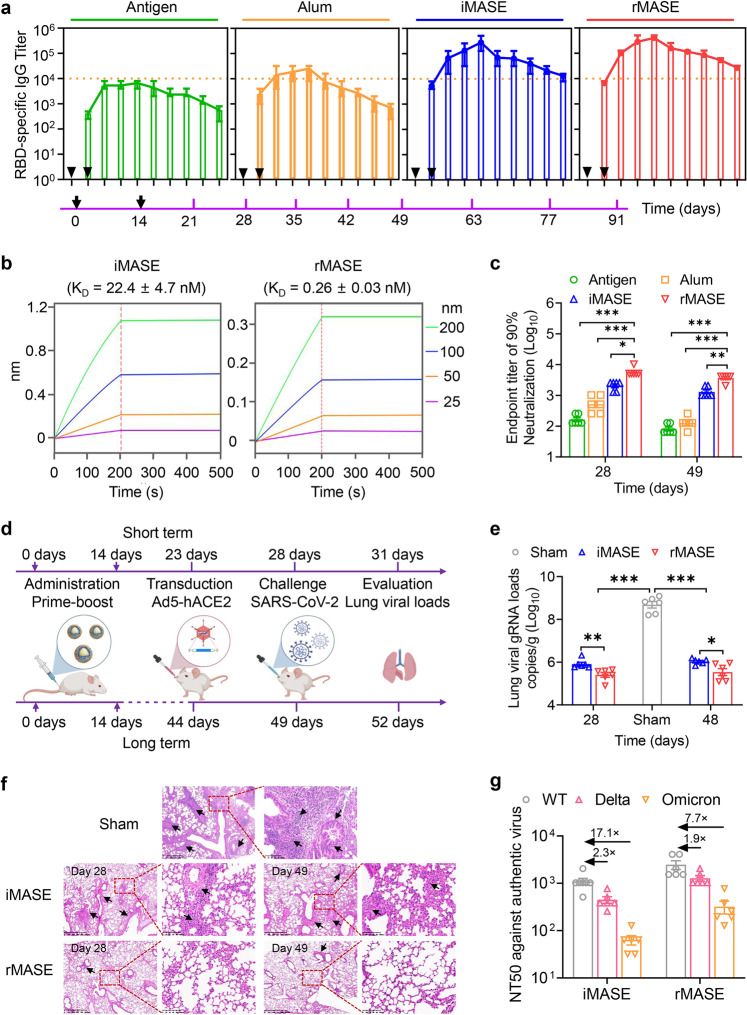
Boosting humoral and cellular response for the persistent protection against SARS-CoV-2. **a** Serum RBD-specific IgG titer over time. Arrows illustrated the time points for vaccinations. The yellow line indicated the highest level of RBD-specific IgG titer induced by Alum. **b** The binding affinity of antibodies with RBD antigen measured by BLI. Post-administration, the antibodies were purified from the mouse serum collected on day 28. The association signals at different concentrations of RBD were monitored and fitted to obtain the kinetic parameters. **c** Measurement of SARS-CoV-2 pseudotyped virus 90% neutralizing titer (NT90) of serum samples from mice) on the 28th and 49th day after the first immunization. The SARS-CoV-2 pseudoviruses were developed by inserting full-length S protein (Wuhan-Hu-1) into vesicular stomatitis virus (VSV) G pseudotyped virus (G*ΔG-VSV). **d** Schematic illustration of the experimental design. BALB/c mice (*n* = 6) were administrated with the indicated formulations at two-week intervals. To assess the short-term immune protection, mice were transduced with 8 × 10^8^ pfu of Ad5-hACE2 via i.n. route on day 23. For long-term evaluations, mice were transduced on day 44. After 5 d, the transduced mice were challenged with 5 × 10^5^ TCID50 of SARS-CoV-2 (hCoV-19/China/CAS-B001/2020, GISAID No. EPI_ISL_514256-7) via the i.n. route, following the harvest of lung tissues to test the viral load and pathology 3 d later. **e** Virus titers in lung. SARS-CoV-2 titration from lung tissue by RT-qPCR probing virus gRNA. **f** Histopathology analysis of the harvested lung tissue. Tissue sections were stained with H&E. The black arrows indicated the infection-related symptoms, including the thickened alveolar walls, vascular congestion, and inflammatory cell infiltration. Scale bar: 625 µm (left) and 100 µm (right), respectively. **g** Serum neutralizing activity evaluated by authentic SARS-CoV-2 mutants, illustrated by the serum half-maximal neutralizing titer (NT50) against live SARS-CoV-2 WT, Delta, and Omicron. The serum was collected on day 28 post-administration. The number represented the fold decrease in neutralizing antibody titer. All data in the graphs were presented as the arithmetic mean ± s.e.m. from three independent experiments. For statistical analysis, a one-way analysis of variance was conducted with Tukey’s correction for multiple comparisons. *P < 0.05, **P < 0.01, ***P < 0.001

Next, RBD-specific humoral response was evaluated (Fig. 6a). rMASE-adjuvanted formulations induced significantly higher RBD-specific IgG titers than Alum (16-fold increase, *P* < 0.001) and iMASE (2-fold increase, *P* < 0.001) after 35 days, and this persisted for longer than 3 months. Then, antibody affinity to the RBD antigen was determined using bio-layer interferometry (BLI). As shown in Fig. 6b and Supplementary Table 6, the association rate constant of rMASE (*K*on = 3.63 × 10^4^ Ms^−1^) was significantly higher than that of iMASE (*K*on = 1.06 × 10^4^ Ms^−1^). Additionally, rMASE elicited a lower value of equilibrium dissociation constant (*K*_D_ = 0.26 ± 0.03 nM) compared with iMASE (*K*_D_ = 22.4 ± 4.7 nM), indicating that reversed delivery of RBD and NP may enhance the antibody affinity against the viral infections. Neutralizing activity against the pseudovirus was subsequently probed. Compared with iMASE, rMASE elicited ~180% and ~275% higher neutralization titers (NT90) on days 28 and 49, respectively (Fig. 6c), which suggested efficient binding capabilities against viral infections. Notably, no differences in NP-specific antibody secretion were observed in either iMASE or rMASE, implying a limited effect of NP on the enhanced neutralizing capability (Supplementary Fig. 16a). The prior delivery of NP was further validated by the elevated cytokine profiles of IL-2, IL-12, TNF-α, and granzyme B (Supplementary Fig. 16b). Furthermore, central memory T cells (CD3^+^ CD8^+^ CD44^high^ CD62L^high^) and effector memory T cells (CD3^+^ CD8^+^ CD44^high^ CD62L^low^) in response to rMASE were significantly increased by 150% (*P* < 0.001) and 148% (*P* < 0.001), respectively (Supplementary Fig. 16c–e), suggesting an increased long-term immune response.^34^

To explore the protective efficacy, a SARS-CoV-2-sensitive animal model was constructed using intranasal transduction of Ad5-hACE2-expressing adenovirus. After 5 d, the transduced mice were intranasally challenged with 5 × 10^5^ TCID50 of SARS-CoV-2 (Fig. 6d).^35,36^ With high serological neutralizing antibody (NAb) titer, rMASE significantly reduced the viral loads in the lung compared with iMASE, with an approximate 1000-fold decrease compared with the sham group (Fig. 6e). No detectable subgenomic RNA (sgRNA) was observed in the rMASE-treated group, indicating that the rMASE vaccine was able to inhibit viral replication in lung tissue (Supplementary Fig. 16f). Furthermore, lung sections from the sham group exhibited thickened alveolar walls, vascular congestion, and inflammatory cell infiltration, which was consistent with viral pneumonia. In addition, iMASE-vaccinated mice exhibited moderate vascular congestion and inflammatory cell infiltration. In the case of rMASE, milder lesions were observed with substantially less infiltration of inflammatory cells (Fig. 6f). Furthermore, pulmonary histopathology was scored based on thickening of the alveolar septa, pulmonary alveolar congestion and inflammatory cell infiltration in the alveoli and trachea. As shown in Supplementary Fig. 16g, high lung lesion scores were found in the control animals, and the scores of lung lesions were reduced in the rMASE-vaccinated animals compared to the iMASE-treated groups. In particular, all six control animals (Sham) showed severe pulmonary alveolar congestion. In contrast, the rMASE-vaccinated group demonstrated almost no signs of alveolar congestion. These results indicated that rMASE can cultivate protective immune responses to diminish SARS-CoV-2-induced infections and lung injuries in mice.

To evaluate the efficacy of rMASE against the prevailing variants, we tested its neutralizing abilities against the live wild-type (WT) and variants of concern Delta (B.1.617.2) and Omicron (B.1.1.529). As shown in Fig. 6g, iMASE experienced a 230% decrease in Delta and a 1710% decrease in Omicron in the NAb titers compared with WT. In the case of rMASE, NAb titers decreased by approximately 2-fold and 8-fold against Delta and Omicron variants, which was less evident than that of iMASE, indicating the enhanced immune protection against the variant infections.

## Discussion

In summary, we developed a multi-layer alum-stabilized emulsion for the inside-out assembly of viral antigens, which led to a higher concentration of NP before the release of the surface antigen. In this manner, rMASE reversed the delivery of the surface antigen and NP and stimulated increased IFN-I-mediated innate immunity, in contrast to the natural package (iMASE). Furthermore, prior engagement of IFN-I signaling boosted adaptive immune responses against the influenza A (H1N1) and SARS-CoV-2 viruses. Thus, without any additional adjuvant components, simply altering the delivery sequence of the surface antigen and NP significantly increased the immune potency and duration against enveloped RNA viruses. Through multi-layer alum-stabilized emulsion, the inside-out strategy may offer a facile and efficient platform to elicit potent vaccine efficiency and broad immune protection.

In addition, the increased immune activations were attributed to the reversed delivery of the core and surface antigens, instead of the positive charges on NP. To test it, RBD and protamine, a commonly employed cationic protein, were loaded sequentially via MASE. As a result, the co-delivery of protamine and RBD failed to increase the immune activations by changing the release sequence of the cargos, which indicated that the inherent positive charges of NP scarcely contributed to the enhanced immune response (Supplementary Fig. 17). Moreover, the adjuvant effect of the inside-out strategy was not restrained by the source or sequence of the NP and surface antigens. Through the sequential delivery with NP, SARS-CoV-2 S1 protein or RBD-monomer as surface antigens also elicited a similar trend via MASE. Additionally, the prior release of NPs from *Escherichia coli* (*E. coli*) and eukaryotic cell lines (baculovirus-insect cells) also induced increased antibody secretion and T cell-mediated immune response (Supplementary Fig. 18).

To further demonstrate the efficacy, rMASE was compared with the commercial adjuvant (AddaVax™), a surfactant-stabilized emulsion in a formulation similar to that of MF59^®^.^37^ As shown in Supplementary Fig. 19, the AddaVax^TM^-adjuvanted formulation failed to elicit comparable IgG titers (*P* < 0.001) and cellular immune responses, indicating increased immune potentiation against the commercial emulsion adjuvant. Subsequently, rMASE was compared with SARS-CoV-2 RBD-mRNA@ lipid nanoparticle (LNP), which shared a similar antigen sequence with the protein RBD antigen employed in this manuscript and formulated with SM-102 as the ionizable lipid, 1,2-distearoyl-sn-glycero-3-phosphocholine (DSPC) as the phosphate lipid, 1,2-dimyristoyl-rac-glycero-3-methoxypolyethylene glycol-2000 (DMG-PEG2000) as the PEGylated lipid, along with the cholesterol.^38^ As shown in Supplementary Fig. 20, rMASE induced slightly lower antigen-specific antibody titers and neutralizing antibody titers against WT than RBD-encoded mRNA@LNP. Additionally, rMASE showed comparable responses to Delta (B.1.617.2) and Omicron (B.1.1.529) variants, and a similar level in the engagement of the IFN-γ-producing T cells. Interestingly, rMASE elicited a higher frequency of central memory T cells (CD3^+^ CD8^+^ CD44^high^ CD62L^high^; *P* < 0.001), suggesting the higher immune memory may be activated by the reversed delivery of the core antigens and surface antigens. By contracting the mRNA-based strategy, rMASE induced comparable immune responses, which were evidently higher than the natural exposure of the antigens (iMASE), indicating that the reversed delivery of antigens may offer an enhanced strategy for recombinant protein vaccinations.

Notably, we do not imply that the inside-out strategy can be applied to all viral vaccines. As a preliminary attempt, we employed the multi-layer alum-stabilized emulsion to deliver the surface antigen (HBsAg) and core antigen (HBcAg) of the hepatitis B virus (HBV, a type of DNA virus). Intriguingly, the inside-out assembly failed to elicit higher immune potency. Instead, it is the natural distribution pattern that entrapped the core antigen inside, but displayed the surface antigen on the outmost layer, which significantly boosted the antigen-specific IgG titers, induced higher levels of IFN-γ^+^ T cells, and increased populations of the central memory T cells (CD44^high^ CD62L^high^) among the CD8^+^ T and CD4^+^ T cells (Supplementary Fig. 21a–d). This may be attributed to the more advanced potency of HBsAg to induce IFN-α expression compared with HBcAg (Supplementary Fig. 21e), which may subsequently potentiate the adaptive immune engagement. Presumptively, for the rational delivery of multi-component vaccines, prior delivery of immunogenic ingredients may result in the enhanced immune effect. As such, the prior delivery of adjuvants, such as Toll-like-receptor agonists and STING activators, before the co-delivered antigens may better boost the immune responses for enhanced vaccinations.

Collectively, the exact replication of live virus may not always offer an optimal solution. In the case of vaccines against H1N1 and SARS-CoV-2, reversed delivery of surface antigens and NPs (core antigens) were proved to potentiate the anti-viral effects. Besides modeling the steric structures of pathogens, it is also imperative to dictate the delivery kinetics of the vaccines, in view of spatiotemporal dynamics during immune activation.

## Materials and methods

### Materials

Alum Hydroxide Gel Adjuvant (10 mg/mL) was purchased from InvivoGen, and squalene was purchased from Sigma. HA and NP of H1N1 influenza virus were purchased from Sino Biological. Recombinant RBD-sc-dimers of spike protein were obtained from the Institute of Microbiology, Chinese Academy of Sciences. The NPs were purchased from VACURE and Sino Biological. Roswell Park Memorial Institute (RPMI) Medium 1640 basic, Dulbecco’s modified Eagle’s medium (DMEM), fetal bovine serum (FBS), and phosphate-buffered saline (PBS; pH = 7.4) were purchased from GIBCO BRL (Gaithersburg, MD, USA.). Fluorescent hydrophilic dyes Cy3, Cy5, and Cy7 were purchased from Fanbo Biochemical Co., Ltd. (Beijing, China). CCK-8 was purchased from Dojindo (Kumamoto, Japan).

HEK293T cells, Huh7 cells, and Vero cells were obtained from the American Type Culture Collection (ATCC). SARS-CoV-2 pseudovirus was obtained from National Institutes for Food and Drug Control (NIFDC). Luciferase 1000 Assay System was purchased from Promega (Madison, WI, USA).

### Animals

All mice were raised in a specific pathogen-free facility, and the Institutional Animal Care and Use Committees at the Institute of Process Engineering, Chinese Academy of Sciences approved all experimental animal protocols (approval ID: IPEAECA2020401 and IPEAECA2021403). This study was performed in strict accordance with the Regulations for the Care and Use of Laboratory Animals and Guideline for Ethical Review of Animals (China, GB/T35892-2018).

### Preparation of rMASE and iMASE

rMASE was prepared through layer-by-layer assembly of alum and antigen on the o/w interface. First, the alum/HA-assembled droplets were prepared by ultrasonication (Branson Digital Sonifier, total time = 120 s, power = 25%, interval time = 4 s) of the mixture of HA, alum, water, and squalene (Sigma, Germany). The alum concentration was 0.5 mg/mL, and the o/w ratio was fixed at 1/9. Then, an additional alum (0.5 mg/mL) was added into alum/HA-assembled droplets and mixed for about 30 min, and subsequently adsorbed with NP (0.05 mg/mL) to constitute the multi-layered alum-stabilized emulsion. Similarly, iMASE was prepared with NP entrapped on the inside, and HA absorbed on the outside.

As a control, an alum-stabilized emulsion was prepared by single-step sonication (total time = 120 s, power = 30%, interval time = 4 s) of alum as the colloidal stabilizers and squalene as the dispersion phase.

### Interactions between outer alum and alum/HA-assembled droplets

QCM-D (Biolin Science/Q-Sense, Sweden) was used to test the interactions between the outer alum and alum/HA-assembled droplets. The chips were modified by spin-coating with alum/HA-assembled droplets, HA and alum-stabilized emulsions, respectively. Once the modified chips were installed, alum (0.5 mg/mL) was added to the QCM chamber at a continuous flow rate of 50 μL/min. The vibration frequency (ΔF) was measured to illustrate the adsorption tendency of the outer alum.

### Characterization of rMASE and iMASE

To characterize the surface topography, the droplets were solidified and observed using scanning electron microscopy (SEM; JEOL, Japan). Briefly, solid alum/HA-assembled droplets were prepared using paraffin wax as the dispersion phase. The multi-layered solidified droplets were generated by cooling the temperature from 50 °C to 4 °C, and the outer alum (0.5 mg/mL) was added to attach for approximately 30 min. The droplets were observed by SEM after appropriate dilution (1:40).

Three-dimensional structured illumination microscopy (3D-SIM; GE Healthcare, Issaquah, USA) and stimulated emission depletion microscopy (STED; Leica, Mannheim, Germany) were used to track the assembly of rMASE. Briefly, the indicated droplets were prepared through the assembly of Cy3-labeled HA, Cy5-labeled NP, and Lumogallion-labeled alum on the droplets. With the diluted droplets (1:50), the assembly steps were observed in detail at ×100 magnification. The surface elemental composition of rMASE was precisely determined using inductively coupled plasma mass spectrometry (ICP-MS; Agilent Technologies, Santa Clara, CA, USA).

### Coverage of the inner antigen and the surface display of outer antigen

The specific binding properties of the antigen and antibody were used to analyze whether the attached alum shielded the inner antigens. To eliminate the non-specific interactions of the antibodies, the formulations were blocked with 4% FBS. The treated droplets were then incubated with anti-HA (Sino Biological Scientific) and anti-NP antibodies (Creative Biolabs). After removing the excess antibodies, Alexa Fluor 647-coupled goat anti-Rabbit IgG (H + L) cross-adsorbed secondary antibody (Thermo Fisher Scientific) and Alexa Fluor 488-coupled goat anti-mouse IgG (H + L) cross-adsorbed secondary antibody (Thermo Fisher Scientific) were added to maintain the reaction at 4 °C for 30 min. After washing off the uncombined secondary antibodies, the droplets were observed under a confocal laser scanning microscope (CLSM; Nikon, Japan).

### Force tendency of the inner and outer antigen

An X-ray diffractometer (XPert3 MRD, X-ray Stress Analyzer, Malvern, UK) was used to measure the residual stress. Data collection was performed using the side-inclination method (Ψ-goniometer). The analysis was performed with an operating voltage of 45 kV and a current of 0.8 mA. The diffraction patterns were obtained using Cu-Kα radiation of wavelength 1.54 Å and an angle from 50° to 60° in steps of 0.02° intervals in the transverse direction. The least-squares method was used to regress each data point into a straight line.

$$M = \frac{{\partial (2\theta )_\psi }}{{\partial \sin ^2\psi }}$$

$$K = \frac{{E_C}}{{2(1 + v)}}\tan \theta _0 \bullet \frac{\pi }{{180}}$$

From the above equation, we measured and calculated the modulus of elasticity E and Poisson’s ratio υ to calculate K, which was combined with the linear slope M to obtain the residual stress equation:

$$\sigma _\varphi = KM$$

Thermal stability was estimated using differential scanning calorimetry (DSC; Netzsch, Germany) to assess the antigens release trend. An empty alum pan was used as the reference. The heating rate of the samples was 10 °C/min in the temperature range of 25–200 °C. The locations of the thermal peaks were determined using GraphPad Prism 9.

### Evaluation of antigen release

To analyze the intracellular alum dynamics, BMDCs were co-incubated with rMASE or iMASE for different periods. In the following step, cells were fixed with 2.5% glutaraldehyde overnight in a 0.5 M phosphate solution. Afterward, cells were postfixed in osmium tetroxide, dehydrated in ethanol, and embedded in Epon. To observe the cells, the dishes were broken into pieces and glued onto Epon sticks, and a range of 80–100 nm was selected for each section. Uranyl acetate and lead citrate were used to stain slices. Transmission electron microscopy (TEM; JEOL, Japan) was performed to obtain images at ×10,000 magnification.

The intracellular antigen release profile was evaluated using a high-content live-cell imaging system (Operetta CLS, PerkinElmer, Waltham, MA, USA). rMASE and iMASE were prepared using FITC-HA and Cy5-NP, respectively, and were co-incubated with BMDCs for 6 h to allow maximum uptake. Residual droplets were removed from the medium. Subsequently, the fluorescence intensities of HA and NP were measured at the indicated times.

### Antigens depot and uptake

To measure antigen retention at the injection site, BALB/c mice (6–8 weeks, female) were intramuscularly administrated the indicated formulations containing 5 µg/dose Cy5-HA conjugate and 5 µg/dose Cy7-NP conjugate (Thermo Fisher). The in vivo imaging system FX Pro (Kodak, Rochester, USA) was used to collect fluorescence signals of the antigens at the injection site after intramuscular administration at the indicated time points.

In addition, we evaluated the uptake in vivo by the intramuscular administration of the indicated formulations to BALB/c mice (6–8 weeks, female). Specifically, muscle tissues at the injection sites were harvested and digested with 0.2% (w/v) collagenase type II for 2 h at 37 °C. Afterward, the cells were collected and blocked with anti-mouse CD16/CD32 antibody. After washing thrice, the cells were stained with Ghost Dye™ Violet 450 Viability, PE dump channel markers (anti-mouse F4/80 and Ly-6C antibodies), and PerCP-Cy5.5 anti-mouse CD11c antibody. Data were collected using flow cytometry (CytoFlex LX; Beckman Coulter).

### Antigen-specific antibody secretion

BALB/c mice (6–8 weeks, female) were intramuscularly administered the indicated vaccines, and serum was collected to detect antigen-specific antibody titers. Briefly, 100 μL of antigen diluent (2 μg/mL) was added to 96-well plates and stored at 4 °C overnight. On the second day, after washing with PBS–0.5% Tween 20 (PBST) three times, the 96-well plates were blocked with 0.5% bovine serum albumin (BSA) at 37 °C for 1 h. The serum was diluted to the appropriate dilution in the plates and incubated at 37 °C for 40 min. Afterward, the HRP-conjugated goat anti-mouse IgG antibody (1:50000; Abcam) was added to react at 37 °C for another 40 min. After washing six times, TMB substrate (50 μL per well) was added, and the reaction was immediately stopped. OD450 values were measured using an Infinite 200 PRO (TECAN, Mannedorf, Switzerland). Antibody titers were defined as the end-point of dilutions where the OD450 value was greater than or equal to twice that of the negative control group. Antigen-specific IgG1 and IgG2a titers were detected on day 28 using the same method.

### ELISPOT evaluations

For ELISPOT evaluation, polyvinylidene difluoride (PVDF)-based membrane plates (Millipore) were activated with 35% ethyl alcohol. Then the plates were coated with anti-mouse IFN-γ or IL-4 antibodies (5 μg/mL) and stored at 4 °C overnight. The next day, the plates were blocked with the culture medium (RPMI 1640, 10% FBS) for more than 1 h. Splenocytes from vaccinated mice were immediately added to the wells at 5 × 10^5^ cells/well and re-stimulated with a specific antigen (2 μg/mL). On the third day, the plates were incubated with the detection antibody (1 μg/mL) for 2 h after removing the cells, followed by incubation with the streptavidin-ALP detection antibody (1 μg/mL) at room temperature. BCIP/NBT was then added to the plates and incubated for approximately 10 min in the dark. Each of the above steps requires washing the plates 3–6 times. Finally, the plates were washed with distilled water at the appropriate time points and air-dried overnight in the dark. The spots were scanned and counted using an ELISPOT Analyzer (AT-Spot 2100, China). Each experiment was repeated three times, and similar results were obtained.

### Influenza A challenge experiments

For the prophylactic protection assay, BALB/c mice (6–8 weeks, female) were randomly divided into different groups and immunized twice by intramuscular injection. After 14 d, the mice were challenged intranasally with influenza A (A/Puerto Rico/8/1934) at a dose of 2 LD50 in 50 μL of PBS. The body weight and survival rate of all mice were monitored daily for 21 d. Mice were euthanized when their body weight loss exceeded 20% of their pre-challenge weight.

BALB/c mice (6–8 weeks, female) were euthanized on day 9 post-challenge to dissect the lung tissues for histology and viral load detection. To measure and quantify viral loads, a section of the lung tissue was homogenized and resuspended in 1 mL TRIzol reagent to extract total RNA for RT-qPCR analysis. The primer pair for the mRNA of the influenza virus was F-5′ AAGACCAATCCTGTCACCTCTGA-3′, R-5′-CAAAGCGTCTACGCTGCAGTCC-3′, and that for the internal reference gene (GAPDH) was F-5′-CAATGTGTCCGTCGTGGATCT-3′, R-5′-GT CCTCAGTGTAG CCCAAGATG-3′.

### Evaluation of the transcriptome

The total RNA of BMDCs co-cultured with iMASE or rMASE for 24 h was extracted using the TRIzol reagent (Invitrogen). RNA libraries were sequenced on an Illumina sequencing platform (Gene Denovo Biotechnology, Co., Ltd., Guangzhou, China). In order to normalize and standardize the expression level of each gene, each part of the transcript per kilobase of transcript per million mapped reads (FPKM) was used. Differentially expressed genes (DEGs) were filtered using FPKM based on the edgeR’s general filtering criteria (log2| fold change | >1, false discovery rate [FDR] < 0.05).

### DC activation at the injection sites

To evaluate DC activation, BALB/c mice (6–8 weeks, female) were intramuscularly administrated with the indicated formulations and euthanized 1-, 3-, 5-, and 7-d post-administration. The tissues at the injection site were selected and immersed in collagenase type II (0.2%, w/v) for 2 h at 37 °C, allowing for the tissue digestion and isolation of the single-cell suspension. Then, the cells were blocked with anti-mouse CD16/CD32 antibody for 20 min and stained with Ghost Dye™ Violet 450, PE-labeled dump channel markers (anti-mouse F4/80 and Ly-6C antibodies), PerCP-Cyanine5.5 anti-mouse CD11c antibody, APC anti-mouse CD40 antibody, and FITC anti-mouse CD86 antibody at 4 °C for 30 min and then analyzed by flow cytometry. Furthermore, the muscle tissues were removed from the injection sites to test the expression of CCR7 among the recruited DCs. The tissues were then transferred to a grinder and homogenized on ice. Afterward, the cells were collected and blocked with anti-mouse CD16/CD32 (mouse Fc block) antibody at 4 °C for 20 min. After washing three times, the cells were stained with Ghost Dye™ Violet 450 Viability Dye, PE dump channel markers (anti-mouse F4/80 and Ly-6C antibodies), and Brilliant Violet 605™ anti-mouse CCR7 antibody at 4 °C for approximately 30 min and then analyzed by flow cytometry (CytoFlex LX, Beckman Coulter).

To determine cytokine secretion at the injection site, approximately 80 mg of the muscle tissue was ground. After removing cell fragments, the supernatant was collected by centrifugation at 500 × *g* for 5 min. The cytokine levels of IFN-α, IFN-β, TNF-α, and IL-2 in the supernatants were determined using enzyme-linked immunosorbent assay (ELISA).

### DC and Germinal center activations in draining lymph node

To analyze the dLN activation, BALB/c mice (6–8 weeks, female) were immunized via intramuscular injection in the calf muscles of the hind limb with 100 μL vaccine that contained 5 µg/dose RBD and 5 μg/dose NP and euthanized at the indicated days. Afterward, the lymph nodes in the popliteal fossa between the biceps femoris and semitendinosus were picked and passed through a 70 μm Cell Strainer (BD Falcon) to obtain single-cell suspensions. In terms of DC subsets in dLN, the obtained single cells were blocked with anti-mouse CD16/CD32 antibody and stained with Ghost Dye^TM^ Red 780, PerCP-Cyanine5.5 anti-mouse CD11c antibody, FITC anti-mouse CD8α antibody, PE anti-mouse CD11b antibody, and PE-Cy7 anti-mouse CD103 antibody. Data were collected by flow cytometry. To detect DC activation, obtained single cells were blocked with anti-mouse CD16/CD32 antibody and stained with Ghost Dye™ Violet 450 Viability, FITC anti-mouse CD11c antibody, and APC anti-mouse CD40 antibody at 4 °C for approximately 30 min, and then analyzed through flow cytometry. In addition, CD40L^+^ T-cells were tested. Briefly, cells were labeled with APC-Cy7 anti-mouse CD3e and eFluor 450 anti-mouse CD40L antibodies and analyzed by flow cytometry.

Tfh and GC B cells were then probed. Briefly, mice were injected intramuscularly with the indicated vaccines and euthanized 7 d post-administration. Afterward, the LN cells were prepared and labeled with the Ghost Dye™ Violet 450, PE dump channel markers (anti-mouse B220, CD11b, CD11c, and F4/80 antibodies) and antibodies against Tfh (PerCP-Cyanine5.5 anti-mouse CD3e, APC-Cy7 anti-mouse CXCR5, and PE-Cy7 anti-mouse ICOS antibodies). To confirm the presence of GC B cells, cells were stained with Ghost Dye™ Violet 450, PE dump channel markers (anti-mouse CD3, CD11c, CD11b, and F4/80 antibodies), and antibodies against GC B cells (FITC anti-human/mouse B220, eFluor^TM^ 660 anti-mouse GL-7, and PerCP-eFlour 710 anti-mouse CD95 antibodies). Antibody dilution was performed for flow cytometry staining according to the manufacturer’s handbook. We defined GC B cells as FAS^+^ GL-7^+^ B220^+^ and Tfh cells as CXCR5^+^ ICOS^+^ CD3^+^.^39,40^ Data were collected by flow cytometry.

Immunofluorescence staining was performed as previously described.^41^ LNs were fixed with paraformaldehyde, embedded in paraffin, and sectioned into slices of 5–6 μm thickness. After deparaffinization and hydration, the samples were washed with Tris-buffered saline containing Tween^®^ 20, treated with 3% H_2_O_2_ for 10 min, and blocked with 2% BSA solution for 30 min. The desired concentration of primary antibody was diluted in 500 µL of 0.1% BSA (CD4, CXCR5, or ICOS), added to the cells, and incubated overnight at 4 °C. The corresponding secondary antibodies were incubated after washing at 1:500 in 2% BSA blocking solution for 1 h at room temperature. After washing, the slides were incubated with 4′-6-diamidino-2-phenylindole in PBS for 5 min. Finally, the autofluorescence was quenched, and the slides were detected using K-Viewer.

Afterward, LN-residing memory B cells were evaluated. Briefly, LNs were collected two weeks after boost immunization. The cells were then stained with Ghost Dye™ Violet 450 Viability, PE dump channel markers (anti-mouse CD3e, CD11c, CD11b, and F4/80 antibodies), FITC anti-mouse B220 antibody, and APC anti-mouse CD27 antibody. Data were collected using flow cytometry.

### Binding affinity measurement

To test antibody affinity, serums from immunized mice were purified using protein A antibody affinity chromatography. The binding affinity of antibodies to the RBD antigen was tested by BLI using the Octet^®^ R8 system (Startorius BioAnalytical Instruments Inc.). Briefly, the biotinylated RBD antigen was diluted to 1200 μL and cured for 600 s. Afterward, the RBD antigen was diluted with PBST to 200 nM and then half-diluted for a total of six concentration gradients (200 nM, 100 nM, 50 nM, 25 nM, 12.5 nM, and 6.25 nM). Antibody samples were added to a 96-well plate, and the assay program was set. Ligand-loaded Streptavidin (SA) biosensors were then incubated with different concentrations of RBD antigen in a kinetics buffer. A global fit of the binding curves generated the best fit with the 1:1 model, and kinetic association and dissociation constants were determined. The data were aligned to obtain the kinetic parameters (*K*_D_) using Fortebio data analysis 12.0.

### Pseudovirus neutralization assay

A published method was used to evaluate the neutralization of SARS-CoV-2. First, the TCID_50_ was determined by infection of Huh7 cells. Afterward, serial dilutions of heat-inactivated serum collected from immunized mice were incubated with 100 TCID_50_ of pseudovirus at 37 °C for 1 h. The mixture was then added to 96-well plates that contained Huh7 and incubated for 24 h. Finally, the cells were lysed, and a Luciferase Assay System (Promega, USA) was applied to evaluate luciferase activity. Experiments were performed according to the manufacturer’s instructions. A relative light unit (RLU) reduction greater than 90% compared with the virus control well was defined as NT90, the highest reciprocal serum dilution at which RLUs were reduced. To determine half of the limit of detection, an NT90 below that level was considered.

### Detection of memory T cells

BALB/c mice (6–8 weeks, female) were euthanized 28 d after the first immunization, and splenocytes were isolated and gently passed through a 100 μm cell strainer (BD Falcon). Next, erythrocyte lysis buffer was added, followed by washing three times with RPMI 1640 to obtain single-cell suspensions. Then, the single-cell suspensions (4 × 10^6^ cells/well) were re-stimulated with the antigen (2 μg/mL) at 37 °C in a humidified, 5% CO_2_ atmosphere. After 48 h of culture, the splenocytes were collected and stained with memory T cell related-fluorescent antibodies, including Ghost Dye™ Violet 450, PE dump channel markers (anti-mouse B220, CD11c, CD11b, F4/80, and Ly-6C antibodies), PE-Cy7 anti-mouse CD3e antibody, APC-Cy7 anti-mouse CD4 antibody, PerCP-Cyanine5.5 anti-mouse CD8a antibody, APC anti-mouse CD44 antibody, and FITC anti-mouse CD62L antibodies. CD44^high^ CD62L^high^ and CD44^high^ CD62L^low^ are known as the central memory T cells and effector memory T cells, respectively. Data were collected by flow cytometry.

### SARS-CoV-2 challenge experiments

For SARS-CoV-2 challenge experiments, BALB/c mice (6–8 weeks, female) were vaccinated with iMASE and rMASE via intramuscular injection on days 28 and 49 before the challenge and compared with the non-immunized mice (term “Sham”). Challenge experiments and immunoassay were performed on the same day under consistent conditions for all the groups. Briefly, mice were intranasally transduced with 8 × 10^8^ vp of Ad5-hACE2 23 or 44 days after the first immunization with iMASE and rMASE to rapidly induce the mouse model of SARS-CoV-2 infection. Five days later, the transduced mice were given 5 × 10^5^ TCID_50_ of SARS-CoV-2 (hCoV-19/China/CAS-B001/2020; GISAID No. EPI_ISL_514256-7) via the intranasal administration,^42^ and all mice were euthanized and necropsied 3 d after challenge. Finally, lung tissues were harvested to calculate the viral loads and detect the pathological status. All animal experiments with SARS-CoV-2 the challenge were operated at the Animal Biosafety Level 3 (ABSL3) facility of IMCAS.

### Determination of viral loads in the lung

For the determination of viral loads in lung tissue samples, RT-qPCR was used. First, the lung tissues of mice were collected and homogenized. Following homogenization and centrifugation, 50 μL of the supernatant was used to extract RNA from SARS-CoV-2 using a MagMAXT^M^ Express Magnetic Particle Processor (Applied Biosystems, USA). RT-qPCR assays were conducted using the FastKing One Step Probe RT-qPCR kit (Tiangen Biotech, China), according to the manufacturer’s instructions.^43^ The N gene of the SARS-CoV-2 genome was detected using the corresponding primers and probes.

### Histopathology analysis

Three days after infection, necropsies were performed on six mice per group following a standard protocol. We collected the lungs of challenged mice, fixed them in 10% neutral buffered formaldehyde, and embedded them in paraffin. Tissue sections (5 μm) were stained with H&E to reflect the characteristics of infection, such as interstitial pneumonitis and alveolitis.

### Live SARS-CoV-2 neutralization assay

For live SARS-CoV-2 neutralization assay, we inactivated the plasma samples collected from mice at 56 °C for 0.5 h. Following serial dilution with cell culture medium at a concentration of 1: 4 or 50,000 ng/mL, the inactivated diluted serum was mixed with 100 TCID_50_ of the SARS-CoV-2 virus and incubated at 37 °C for 1 h. Next, the mixtures were added to the 96-well plates covered with confluent Vero cells. Next, the mixture was incubated for another 5 d at 37 °C. Three different individuals were observed, and the cytotoxic effect (CPE) of each well was recorded under a microscope. The CPE was then used to calculate neutralizing titers using the Reed Muench method. All experiments were conducted at the biosafety level 3 (BSL3) facility of the SINOVAC.

### Statistics

All animal studies were performed after randomization. All values are expressed as the mean ± the standard error of the mean (s.e.m). Data were analyzed by one- or two-way analysis of variance (ANOVA) for comparison of multiple groups using GraphPad Prism 9 and Origin 9 software. Flow cytometry data were analyzed using FlowJo 10.0 and CytExpert Software 2.3. Statistical significance was set at a *P*-value less than 0.05 (*P* < 0.05).

## Supplementary information

Supplementary Materials

## Data Availability

All data or resources used in the paper are available by reasonable requirements to the leading correspondence, Prof. Guanghui Ma (ghma@ipe.ac.cn).
