# Supplementary material for: Inside-out assembly of viral antigens for the enhanced vaccination
Source: Signal Transduct Target Ther. 2023 May 24;8:189. doi: 10.1038/s41392-023-01414-7 (PMC10205815; doi:10.1038/s41392-023-01414-7)
Supplement: Supplementary file 1 — Supplementary Materials [file 41392_2023_1414_MOESM1_ESM.docx]

Supplementary Materials for

**Inside-out assembly of viral antigens for the enhanced vaccination**

Fengqiang Cao^1, 3, 4^, Sha Peng^1, 3, 4^, Yaling An^5, 6^, Kun Xu^5, 7^, Tianyi Zheng^5, 8^, Lianpan Dai^5^, Kenji Ogino^3, 4*^, To Ngai^9^, Yufei Xia^1, 2, 10*^, Guanghui Ma^1, 2, 4, 10, 11*^

Correspondence to: Yufei Xia (yfxia@ipe.ac.cn), Guanghui Ma (ghma@ipe.ac.cn) and Kenji Ogino (kogino@cc.tuat.ac.jp)

**This PDF file includes:**

Figures. S1 to S21

Tables S1 to S6

**
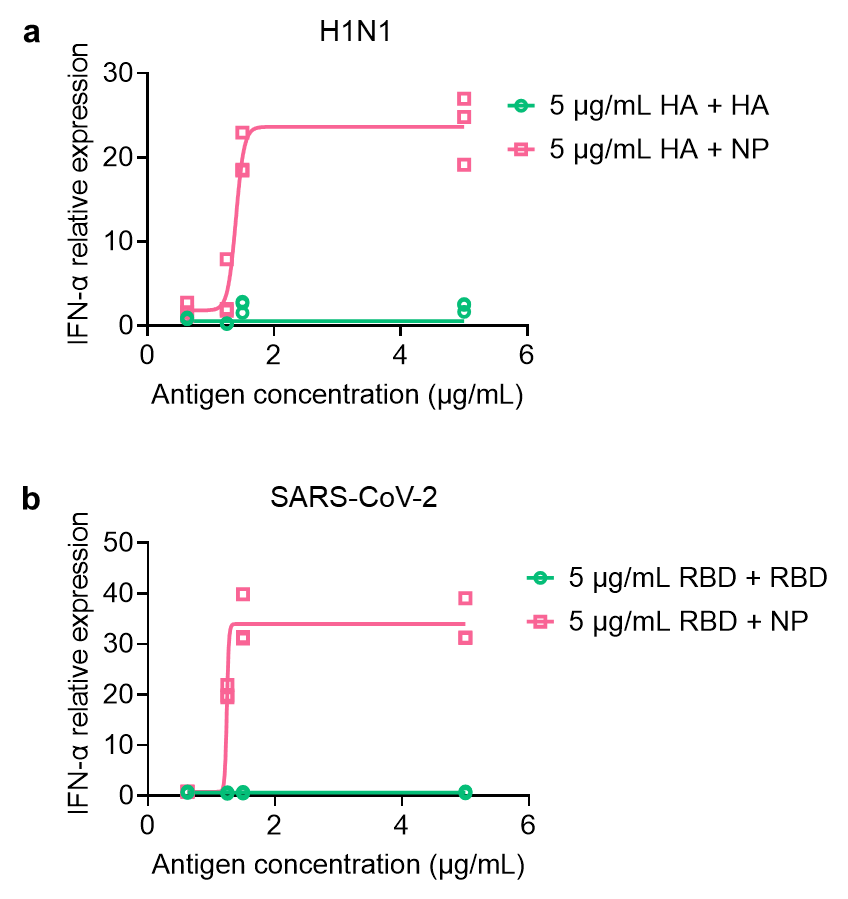
**

**Supplementary Fig. 1. The expression of IFN-α by the BMDCs after surface antigen and nucleocapsid protein of (a) H1N1 influenza virus and (b) SARS-CoV-2 treatment.**

For H1N1, HA and NP were used to treat BMDCs, the mRNA expression of IFN-α was determined using RT-qPCR. These data were analyzed by nonlinear-regression (dose-response-stimulation).

Simply increasing the levels of the surface antigens barely boosted the IFN-α expression. However, the IFN-α levels were in accordance with the presence of NP. With 2 μg/mL NP in the system, evident IFN-α-relative gene expression was observed, indicating that the pre-exposure of the NP before the surface ones may confer the enhanced IFN-I-mediated immune response and anti-viral effect.


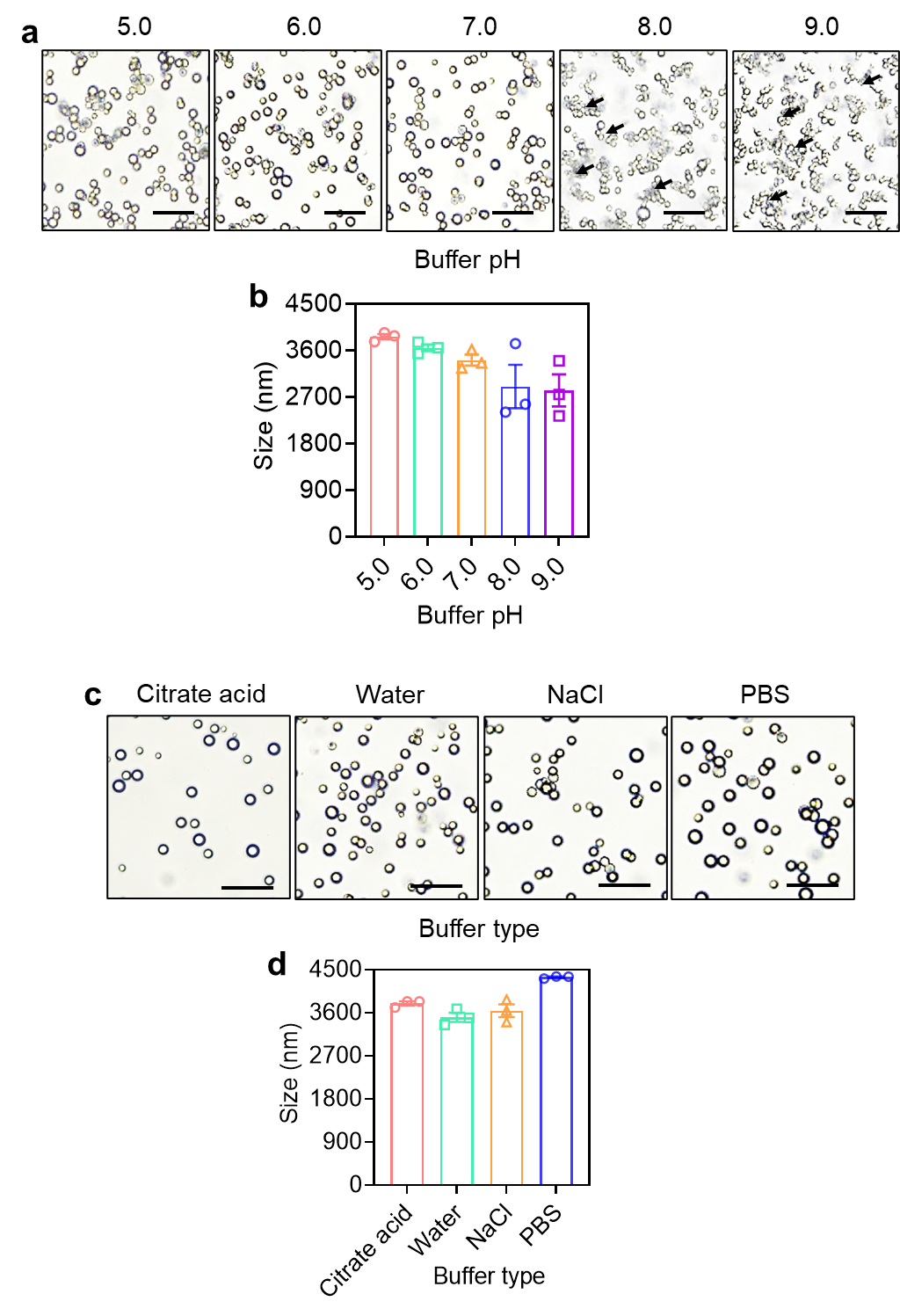


**Supplementary Fig. 2.** **Optimization of the buffer pH and buffer type for alum/HA-assembled droplets.**

To harness the delivery kinetics of antigens, surface antigen was trapped within the nanocage through the co-assembly of alum and antigen on the oil/water (o/w) interface. In this research, to meet the requirements of safety and immunization strategies, the 0.5 mg/mL alum was selected as stabilizer. To simulate the formulations of oil emulsion adjuvants, the squalene was selected as the dispersion phase. Alum/HA-assembled droplets were evaluated after 3 days of preparation. **(a)** Optical micrographs and **(b)** size distributions of droplets constructed by water with different pH. **(c)** Optical micrographs and **(d)** size distributions of droplets constructed with different buffers. The optical images were acquired with 20 × magnification. Scale bar: 20 μm. The black arrows indicated the flocculation of droplets. The droplet sizes were determined by dynamic light scattering (DLS, Malvern) via nano zeta sizer. Data were shown as mean ± s.e.m. (n = 3, from 3 independent experiments).

With similar compositions, smaller droplets may harbor higher interfacial energy for enhanced stability. For the buffer pH, although the smallest emulsion was obtained in buffer with pH 8.0-9.0, evident flocculation of droplets was observed, which was not conducive to the deposition of the outer alum. In comparison, buffer with pH 7.0 could obtain homogeneous droplets, which might be the optimum for a continuous phase. Furthermore, citrate acid, water, NaCl, and PBS were used as a continuous phase to prepare alum/HA-assembled droplets, respectively. As shown in the results, citrate acid, PBS, and NaCl as continuous phase obtained biggish emulsion droplets, which may be affected by the charge screening effect of saline ions. Only water as a continuous phase met requirements of both smaller size and homogeneity. Therefore, water (pH = 7.0) may be the optimal continuous phase.


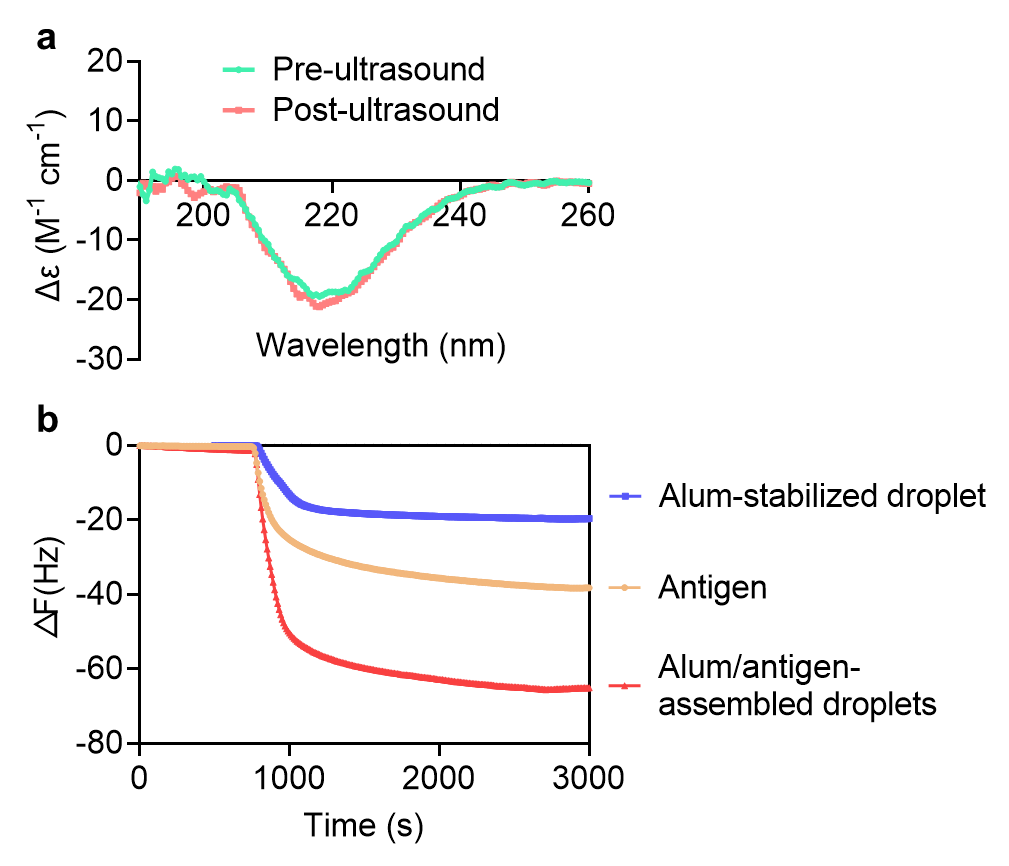


**Supplementary Fig. 3. The characterization of alum/HA-assembled droplets.**

**(a)** The circular dichroism analysis of the HA before and after the co-assembly with alum on the o/w interface. To determine the secondary structure of the antigen after assembly, the droplets were demulsified and the HA was collected by dialysis method (100 kDa MWCO, Millipore). The spectra were detected via a JASCO spectrometer, where the X axis showed wavelength in nm and the Y axis showed molar ellipticity per residue ([θ], deg cm^2^ dmol^-1^). **(b)** Adsorption of alum on alum/antigen co-assembled droplets. The adsorption of alum on the alum/antigen co-assembled droplets (the 2nd layer of the droplet) was analyzed via quartz crystal micro-balance with dissipation monitoring (QCM-D, Q-SENSE E4). The chips were modified with HA, alum-stabilized emulsions, and alum/HA-assembled droplets, respectively, and then interacted with the fluidic alum in a continuous mode (flow rate = 50 μL/min). The stronger $\Delta F$ (red line) indicated the increased adsorption of alum on alum/antigen assembled droplets.

**
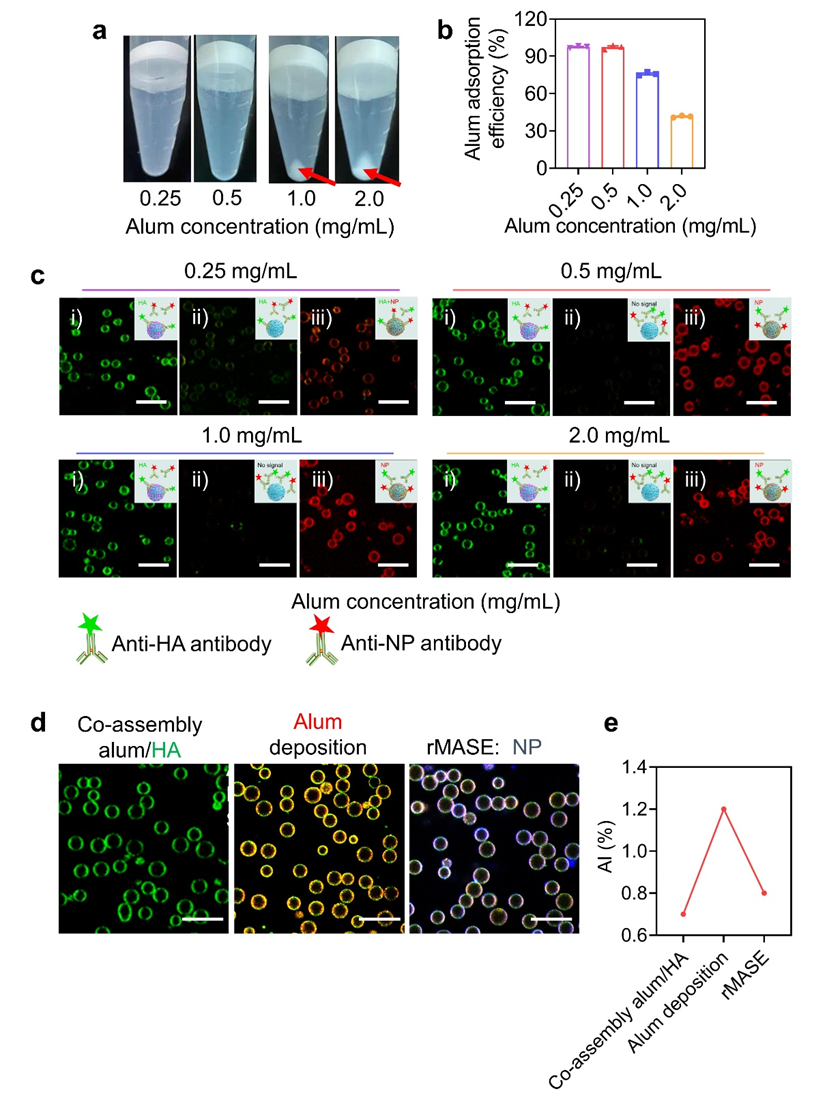
**

**Supplementary Fig. 4. Optimization of alum concentrations for the stable multi-layer droplets.**

To obtain multi-layered droplets, the fluidic alum was added and mixed with the alum/HA-assembled droplets for 0.5 h. **(a)** Appearance of the droplets adsorbed various concentration of alum after high-speed centrifugation (6000 g, 10 min). **(b)** Alum adsorption efficiency on the alum/HA-assembled droplets. Alum adsorption efficiency (%) = (Alum input - Fluidic alum after mixing) / Alum input × 100%. **(c)** Verifying the coverage of the inner HA and the surface display of NP for the inside-out strategy. The droplets were treated with 4% (v/v) FBS solution to avoid non-specific interactions and then treated with a mixture of anti-HA and anti-NP antibodies, followed by a confocal imaging. Scale bar: 10 µm. **(d)** Confocal image of rMASE. HA, NP and the outer alum were labeled with Cy3 (green), Cy5 (blue), and lumogallion (red), respectively. The images were acquired with 100 × magnification via confocal laser scanning microscope (CLSM). Scale bar: 10 μm. **(e)** Atomic percentages of aluminum element on the droplets surface were determined via inductively coupled plasma mass spectrometry (ICP-MS). Data of **(b)** was shown as mean ± s.e.m. (n = 3, from 3 independent experiments).

As shown in Supplementary Fig. 4, as alum/HA-assembled droplets were mixed with 0.5 mg/mL alum, the inner antigens were thoroughly shielded, and there was no redundant alum remained in continuous phase. 0.25 mg/mL alum could not thoroughly cover the inner antigen, while the excess alum remained in a continuous phase when 1 mg/mL alum was added. Therefore, 0.5 mg/mL alum may be suitable as the outer layer on the alum/HA-assembled droplets.


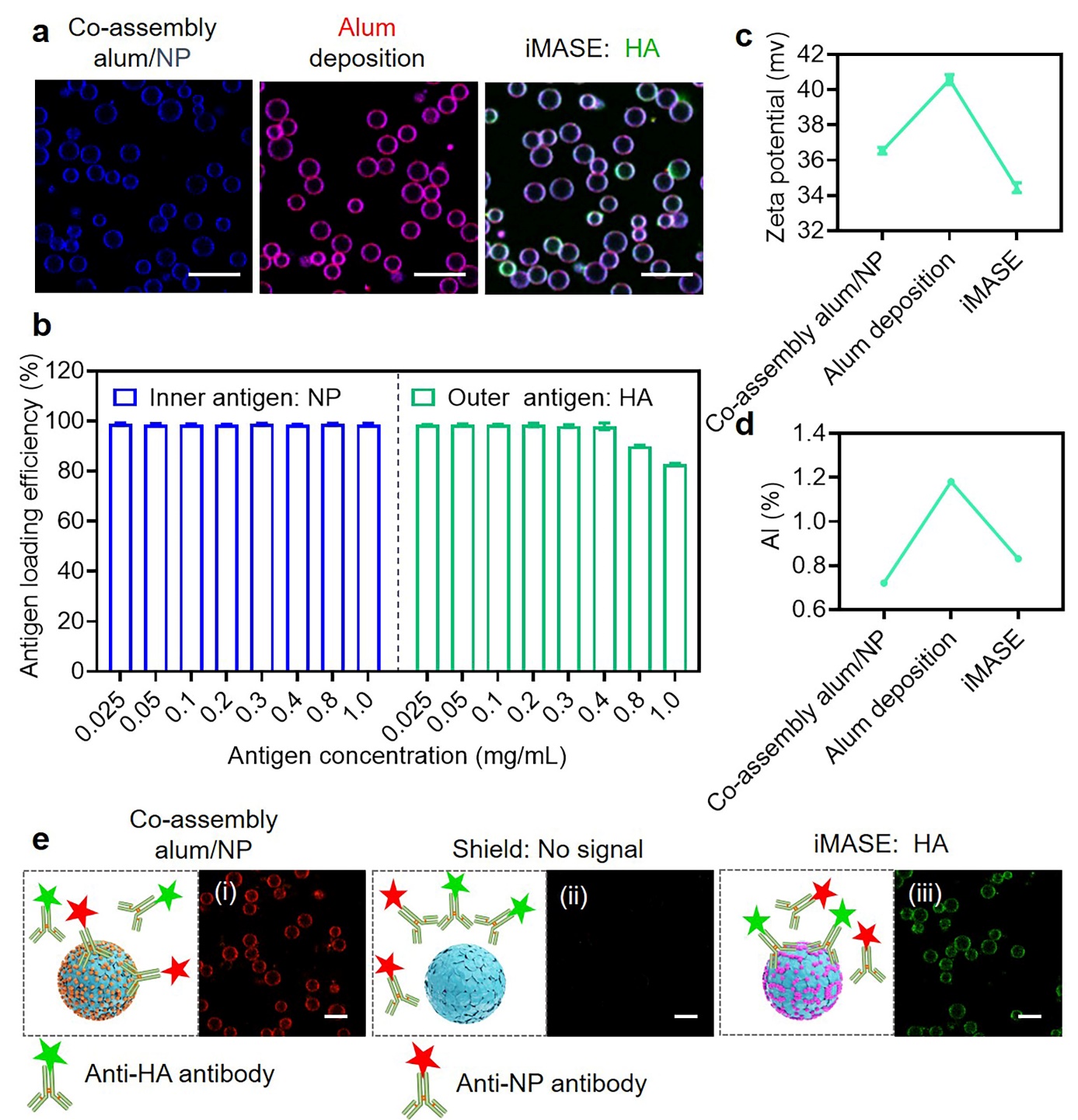


**Supplementary Fig. 5. Characterization of the optimized iMASE.**

**(a)** Confocal image of iMASE. HA, NP, and outer layer of alum were labeled with Cy3 (green), Cy5 (blue), and lumogallion (red), respectively. The images were acquired with 100 × magnification via CLSM. Scale bar: 10 μm. **(b)** The antigen loading efficiency of HA and NP. The antigen loading efficiency was determined using the equation: Antigen loading efficiency

(%) = (Total antigens - Fluidic antigens) / Total antigens × 100%. **(c)** Zeta potential of iMASE. The zeta potential of droplets was determined by DLS. **(d)** The atomic percentage of aluminum element on the droplets surface were determined via ICP-MS. **(e)** Verifying the coverage of the inner NP and the surface display of HA for the iMASE. After treating the droplets with 4% (v/v) FBS solution to avoid non-specific interactions, the droplets were incubated with the mixture of anti-HA antibody (green) and anti-NP antibody (red), followed by confocal imaging. The confocal images were acquired with 100 × magnification. Scale bar: 5 μm. Data of **(b)** and **(c)** were shown as mean ± s.e.m. (n = 3, from 3 independent experiments).

iMASE was prepared to load NP on the inside and adsorb HA on the outside of the droplets, which were determined with similar size and antigen loading efficiency compared with rMASE. The size distributions of droplets were uniform. Tracing by the changes of zeta potentials and element compositions, iMASE demonstrated the natural antigen distributions of HA and NP. Furthermore, there was an evident reduction in the fluorescent intensity of NP after adsorbing the outer alum, suggesting the NP covered by outer alum to avoid pre-exposure during the vaccine delivery. Additionally, after loading with the outer HA, only the fluorescent signal of HA was observed, indicating the surface display of the HA.

**
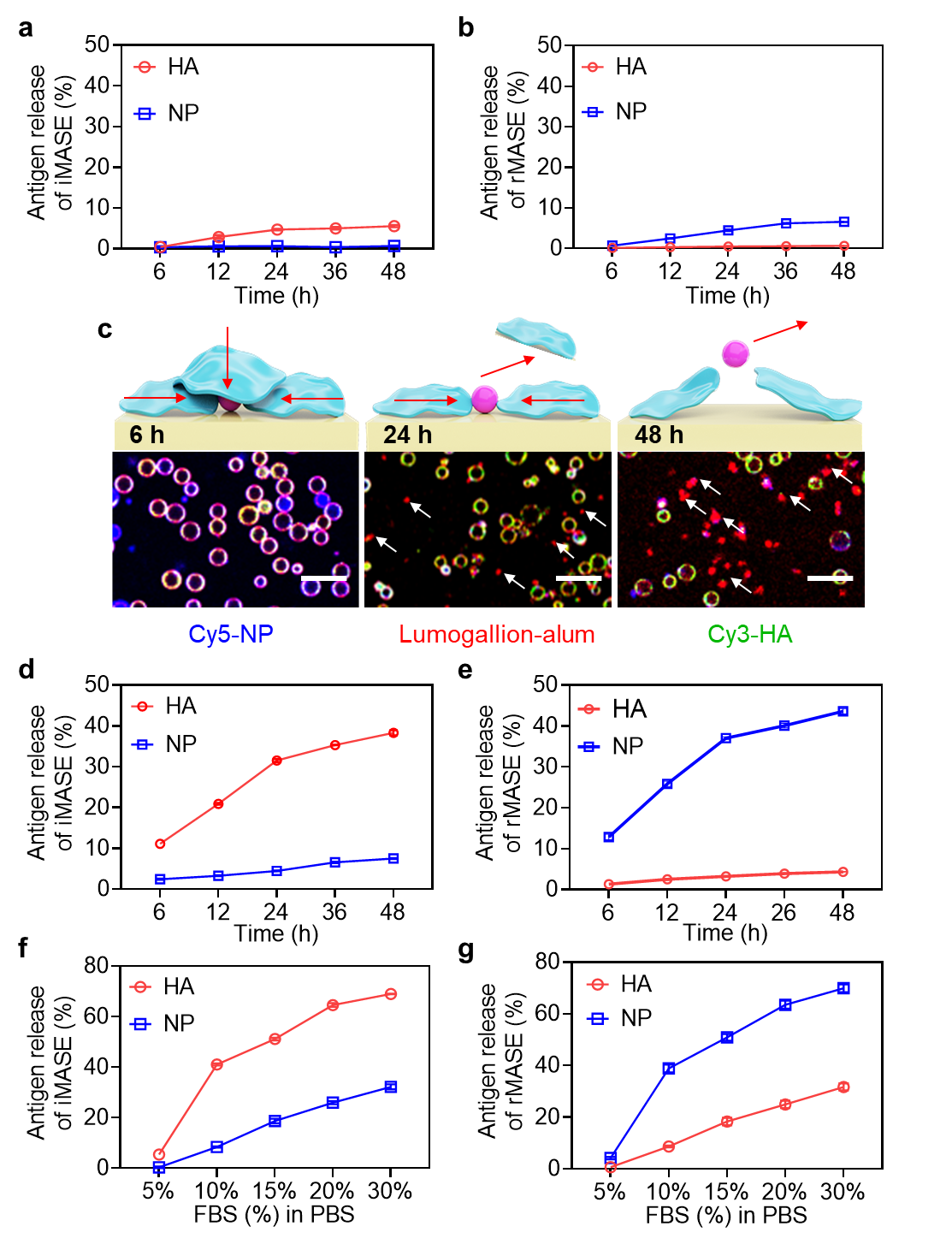
**

**Supplementary Fig. 6.** **The release profiles of iMASE and rMASE.**

The release profiles of HA and NP from **(a)** iMASE or **(b)** rMASE after incubation in the simulated interstitial fluid for the indicated time periods. The simulated interstitial fluid was prepared by removing the macromolecules that were larger than 30 kDa from 10% (v/v) FBS in PBS. **(c)** Confocal images of rMASE after incubation with the 10% (v/v) FBS in PBS. HA, NP, and alum were labeled with Cy3 (green), Cy5 (blue), and lumogallion (red), respectively. The white arrow indicated the scattering of alum that was associated with antigen release. Scale bar: 10 μm. The release profiles of **(d)** iMASE and **(e)** rMASE in 10% FBS (v/v) in PBS. The release profiles of **(f)** iMASE and **(g)** rMASE in the climbing FBS concentrations at 48h. HA and NP were labeled with FITC and Cy5, respectively. The fluorescence intensity of fluid antigen was determined by fluorescence spectrophotometer. Antigen release was determined by fluorescence intensity of fluid antigen/fluorescence intensity of total antigen × 100%. Data were shown as mean ± s.e.m. (n = 3, from 3 independent experiments).

Limited antigens were released from iMASE and rMASE under the simulated interstitial fluid, indicating that the antigens can hardly discharge from the droplets before entering the cells.

In the case of rMASE, the outer antigen (blue) began to release along with the detachment of the outer alum after incubation with the 10% (v/v) FBS in PBS. Approximately 31.0% of the NP were released within 24 h. Over time, an apparent dissociation of alum was observed, and the inner antigen (green) was discharged from the droplets subsequently. With the shielding of the alum on the interface, HA release was quite slow. Additionally, a reversed release trend in iMASE was observed. Therefore, the consecutive loading of HA and NP on the iMASE and rMASE could result in the release of the outer antigen before the inner antigen. Furthermore, the release rates were increased with the climbing of the FBS concentrations, suggesting that antigens were discharged by the ligand exchange of the macromolecular proteins.


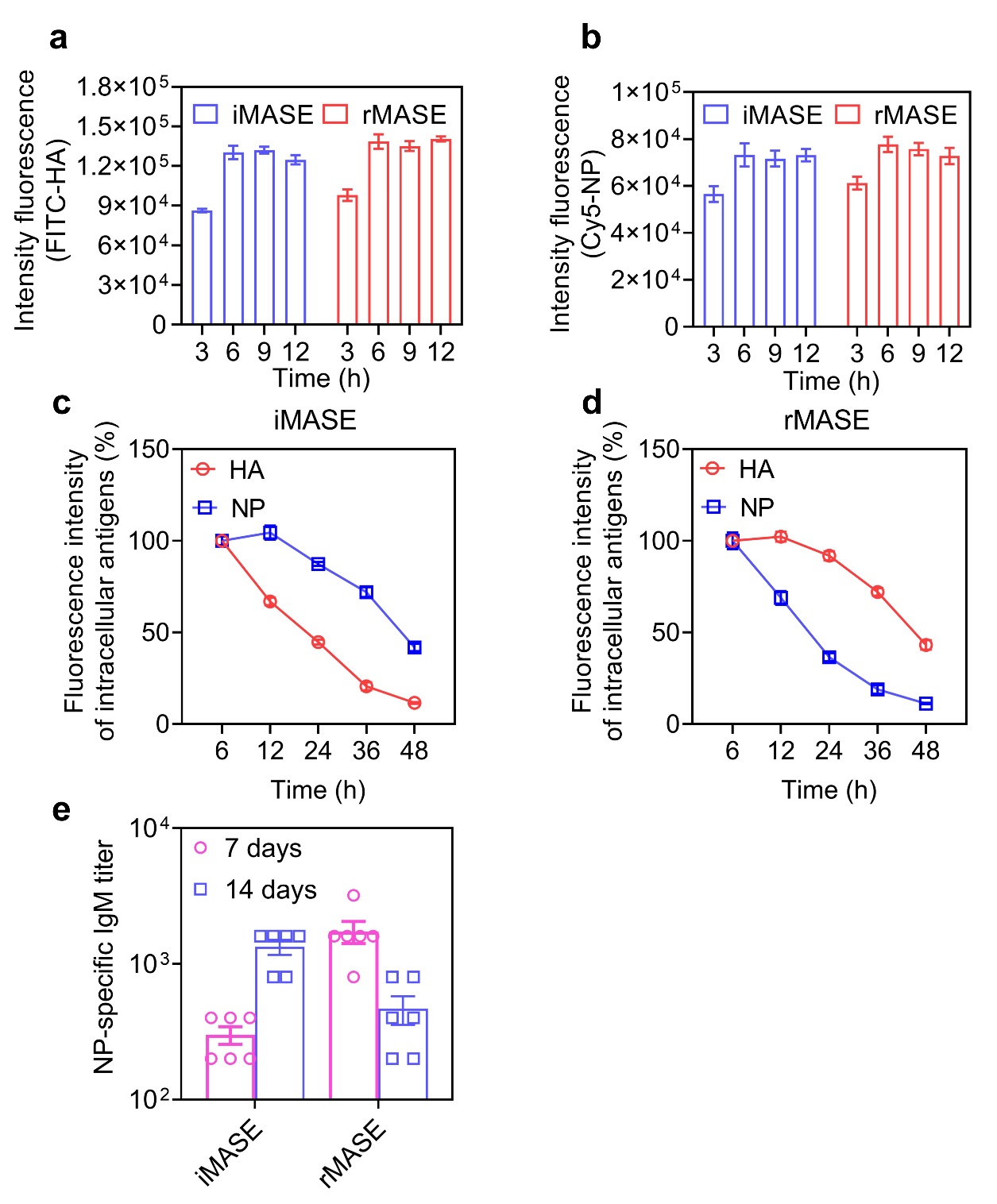


**Supplementary Fig. 7. The delivery kinetics of antigens *in vitro* or *in vivo*.**

Internalization of **(a)** HA and **(b)** NP by BMDCs at indicated time points. Flow cytometry on the uptake of HA and NP after co-incubation with rMASE and iMASE. HA and NP were labeled with FITC and Cy5, respectively. The quantitative analysis of the fluorescent intensity of HA (red line) and NP (blue line) within the **(c)** iMASE-treated BMDCs and **(d)** rMASE-treated BMDCs. HA and NP were labeled with FITC and Cy5, respectively. And the average fluorescent intensity of antigens was detected by high content imaging system (Perkin Elmer) at 6 h, 12 h, 24 h, 36 h, and 48 h. Data were calculated by the equation: Fluorescence intensity of intracellular antigen (%) = (Average fluorescence intensity at indicated times/Average fluorescence intensity at 6 h) ×100%. **(e)** NP-specific IgM titers on day 7 and day 14 post-administration. Data were shown as mean ± s.e.m. (n = 6, from 3 independent experiments).

Compared to the fluorescent enrichment via the micro-sized droplets, the fluidic antigens were too weak to be detected. Accordingly, the release profile can be assessed by comparing the fluorescence decay of the loaded antigen. For rMASE, the fluorescence intensity of NP was decreased by ~35%, but the fluorescence intensity of HA remained constant at 12 h. Additionally, there was a limited fluorescent signal for NP, but about 40% of total fluorescence intensity was preserved for HA after 48 h incubation. In the case of iMASE, the fluorescence of NP was not evidently reduced until 24 h. Accordingly, these data demonstrated that rMASE, through loading HA on the inside and NP on the outside, enabled the prior release of the outer NP within the BMDCs, which may reverse the cellular exposure sequence of H1N1 antigens.

As compared to the serum on day 7, rMASE-pulsed mice experienced a decreased NP-specific IgM titer on day 14 after intramuscular injection. Contrasting to iMASE, NP induced evidently boosted NP-specific IgM titer on day 14, indicating the lagged release of the inner antigen.


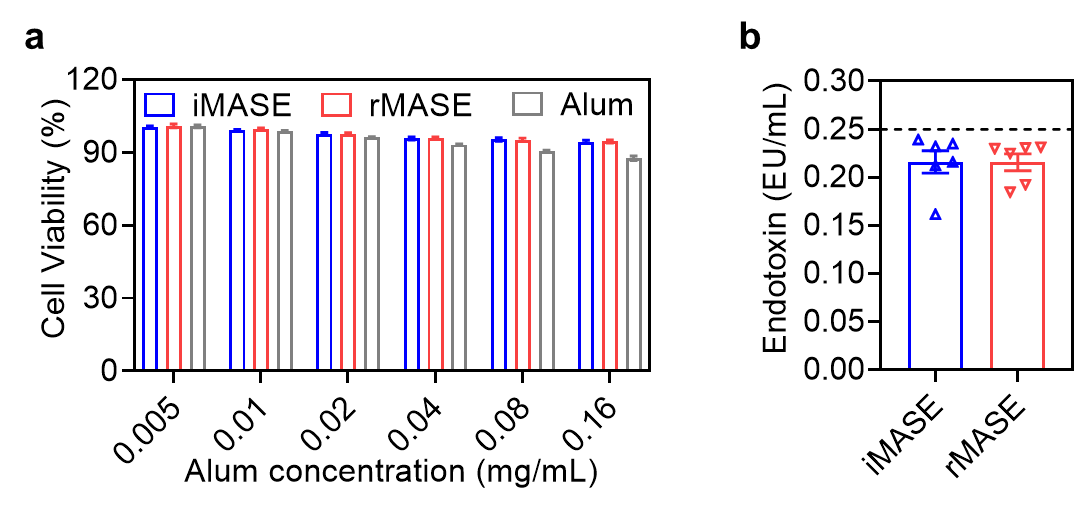


**Supplementary Fig. 8. Cytotoxicity and endotoxin levels of the testing groups.**

**(a)** Cytotoxicity performed on BMDCs. The dose effect of iMASE and rMASE were demonstrated by the alum concentrations in the system. Cell viability was assessed using the colorimetric cell counting kit-8 (CCK-8) from Dojindo Laboratories (Kyoto, Japan) at 48 h. **(b)** Endotoxin level of the formulations. Endotoxin levels were detected via the chromogenic LAL endotoxin assay kit (GenScript, USA). Data were shown as mean ± s.e.m. (n = 6, from 3 independent experiments).

For both iMASE and rMASE, limited cytotoxicity was observed under the droplet concentrations below 0.16 mg/mL. All the tested formulations remained in a relatively lower range of endotoxin level (0.25 EU/mL), which indicated that endotoxin contamination hardly influenced the enhanced immune responses. These data suggested that the increased DC activation was attributed to exposure of NP before HA instead of the potential material contamination or cell damage.


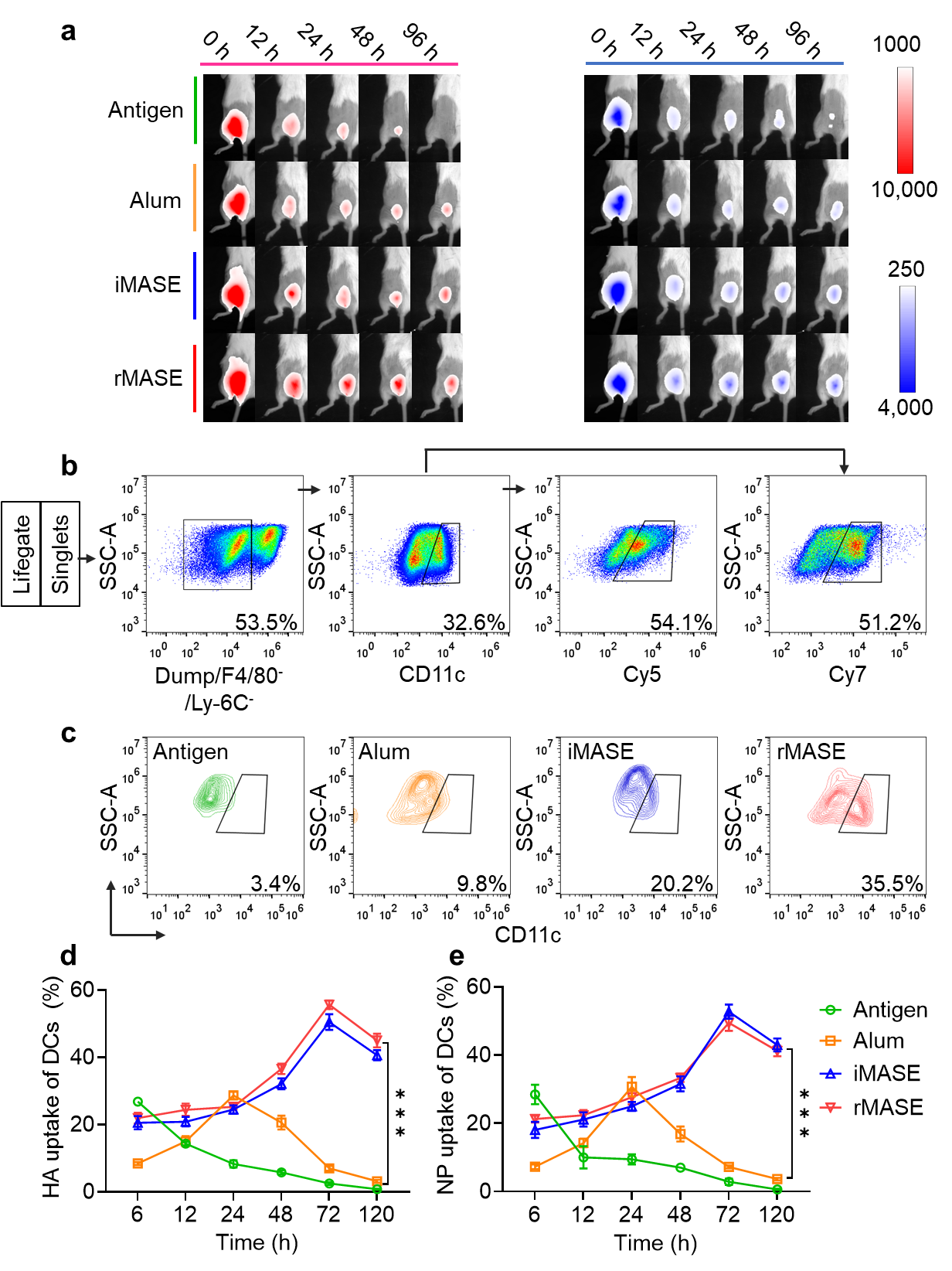


**Supplementary Fig. 9. Antigen depot and uptake at the injection site.**

**(a)** Fluorescent intensity of antigen persistence over time. HA (left, red) and NP (right, blue) were labeled with Cy5 (ex: 648 nm; em: 662 nm) and Cy7 (ex: 743 nm; em: 767 nm), respectively. BALB/c mice (n = 6) were intramuscularly administrated with the indicated formulations (5 μg HA and 5 μg NP in 100 μL per mouse). Antigen persistence at the injection site was measured by *in vivo* imaging system FX Pro (Kodak) at the indicated time points. **(b)** Gate strategy for antigen uptake at the injection site. **(c)** Flow cytometry analysis on DC recruitment. Flow cytometry analysis on **(d)** HA and **(e)** NP uptake by DCs at the injection site. HA and NP were labeled with Cy5 and Cy7, respectively. BALB/c mice (n = 6) were intramuscularly administrated with the indicated formulations. The single-cell suspension was prepared to analyze the recruitment and antigen uptake of DCs. All data in the graphs were presented as arithmetic means ± s.e.m. from three independent experiments. For statistical analysis, a two-way analysis of variance was carried out with Tukey’s correction for multiple comparisons. **P* < 0.05, ***P* < 0.01, ****P* < 0.001.

Compared with other groups, rMASE significantly increased DC recruitments at the injection site. Furthermore, iMASE and rMASE demonstrated a similar trend in DC internalization, indicating that the sequential release happened intracellularly.


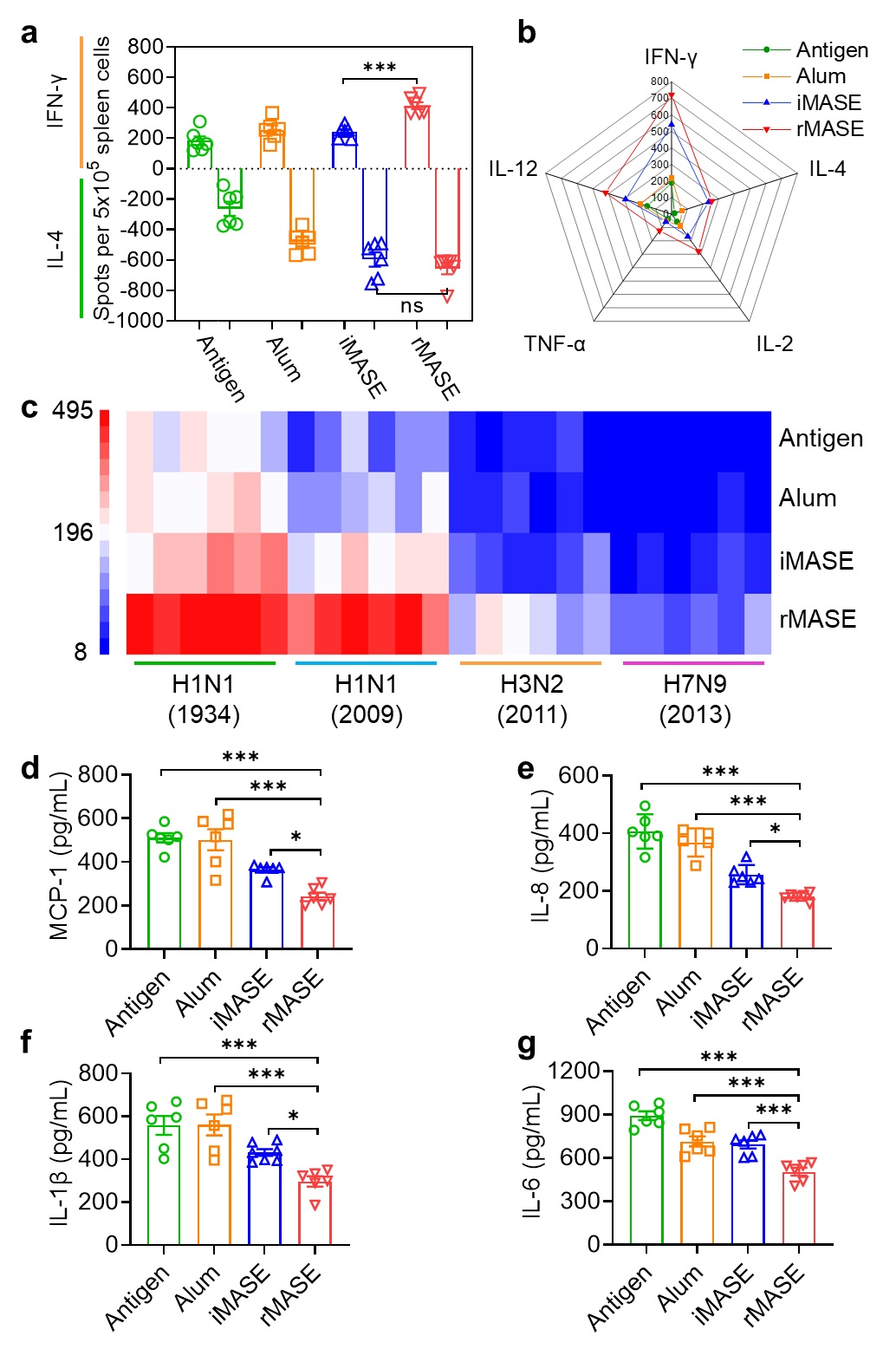


**Supplementary Fig. 10. The enhanced immune response against the H1N1 influenza virus.**

**(a)** Frequency of IFN-γ and IL4-secreting cells among splenocytes via ELISPOT assay. ELISPOT analysis of IFN-γ and IL-4 spot-forming cells among the splenocytes after *ex vivo* restimulation with HA on day 28. **(b)** Comparison of the secretion of cytokine (IL-2, IFN-γ, TNF-α, IL-4, and IL-12) in the supernatant of the *ex vivo* stimulated splenocytes. ELISA analysis of the cytokines from the splenocytes after *ex vivo* restimulation with HA on day 28. **(c)** Heatmap representing the frequency of IFN-γ-producing cells responding to different HA antigens. ELISPOT analysis of IFN-γ spot-forming cells among the splenocytes after *ex vivo* restimulation with different HA on day 28. Blue boxes correlate with lower cytokine present and red boxes with higher cytokine present (see scale). The levels of **(d)** monocyte chemoattractant protein 1 (MCP-1), **(e)** interleukin-8 (IL-8), **(f)** interleukin-1β (IL-1β), and **(g)** interleukin-6 (IL-6) in lung homogenates were detected by ELISA after viral challenge. All data in the graphs were presented as arithmetic means ± s.e.m. from three independent experiments. For statistical analysis, a one-way analysis of variance was carried out with Tukey’s correction for multiple comparisons. **P* < 0.05, ***P* < 0.01, ****P* < 0.001.


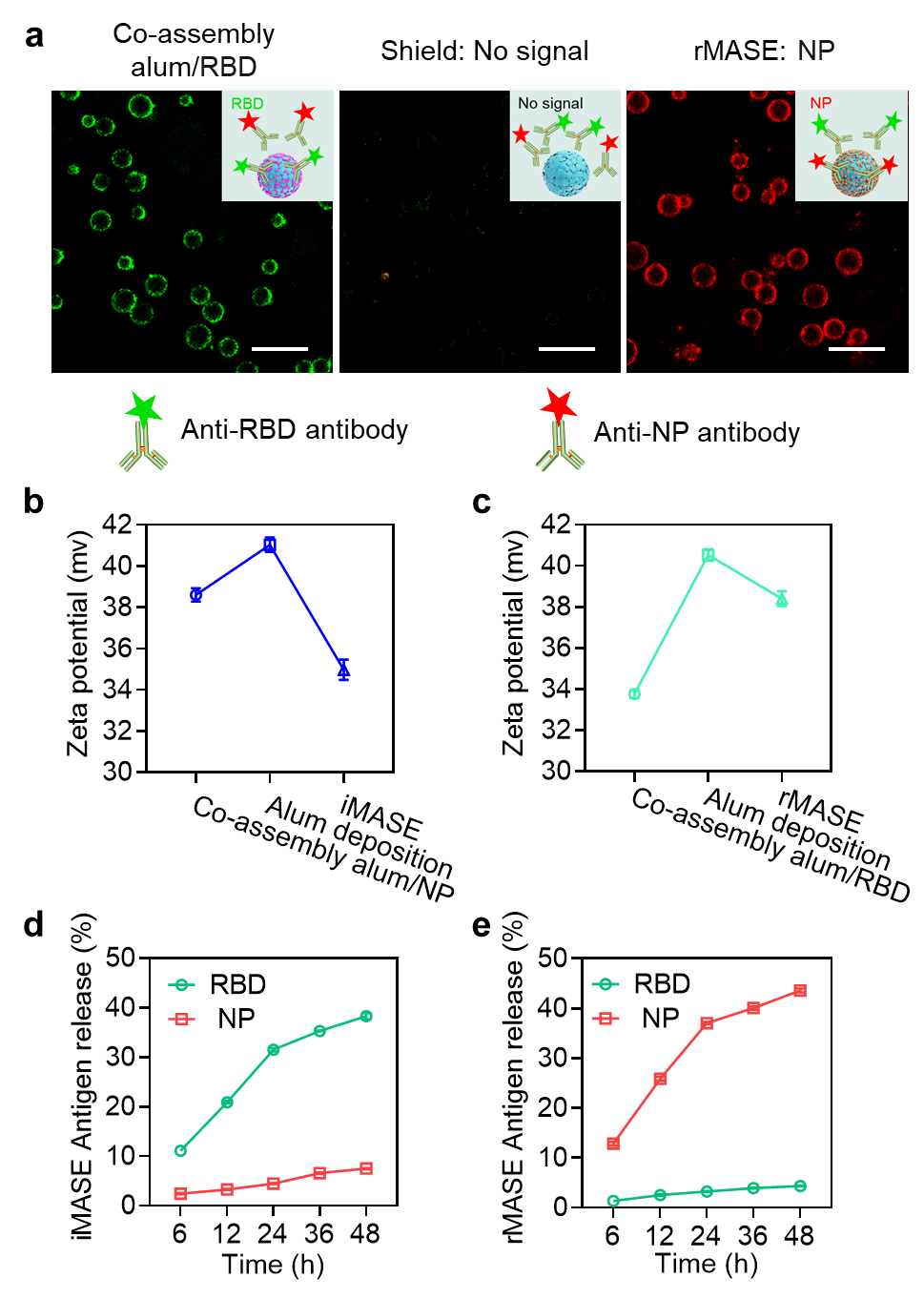


**Supplementary Fig. 11. Inside-out assembly of RBD and NP.**

**(a)** Verifying the coverage of the inner RBD and the surface display of NP for the inside-out strategy. The droplets were treated with 4% (v/v) FBS solution to avoid the non-specific interactions and then treated with a mixture of anti-RBD and anti-NP antibodies, followed by confocal imaging. Scale bar: 10 µm. Zeta potential of **(b)** iMASE and **(c)** rMASE. **(d)** The release profile of iMASE in FBS (10% v/v in PBS). **(e)** The release profile of rMASE in FBS (10% v/v in PBS). Data were shown as mean ± s.e.m. (n = 3, from 3 independent experiments).

Following the assembly of RBD and NP, the zeta potential was changed, indicating the multi-layer alum-stabilized emulsion achieved successive loading of the viral antigens. As shown in Supplementary Fig. 11a, the RBD was shielded by the outer alum, with the display of NP on the surface of the droplets. For the release profile, high concentration of NP (outer antigen, red) began to release within 24 h in the rMASE group. In contrast, limited RBD (inner antigen, green) was released within 48h. Additionally, a reversed release trend in iMASE was observed, which achieved higher release concentrations of RBD before NP. Therefore, the consecutive loading of RBD and NP on the iMASE and rMASE could result in the release of the outer antigen before the inner antigen.


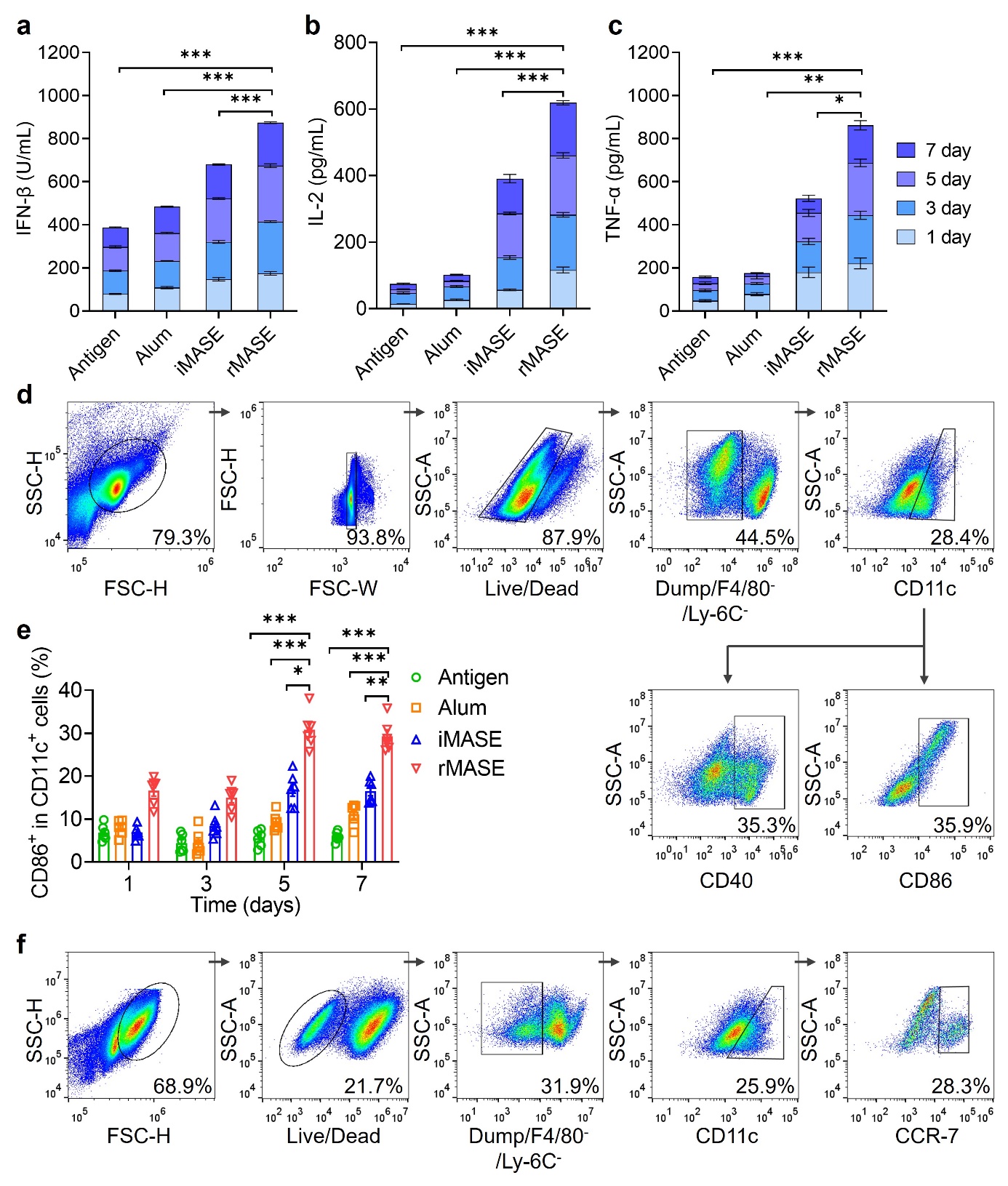


**Supplementary Fig. 12. The local immune reactions at the injection site.**

The secretions of **(a)** IFN-β, **(b)** IL-2, and **(c)** TNF-α at the injection site. **(d)** Gate strategy for CD86 and CD40 subsets among the recruited DCs. **(e)** Flow cytometry on the expression of CD86. **(f)** Gate strategy for CCR-7 subsets among the recruited DCs. All data in the graphs were presented as arithmetic means ± s.e.m. A two-way analysis of variance was carried out with Tukey’s correction for multiple comparisons. **P* < 0.05, ***P* < 0.01, ****P* < 0.001.


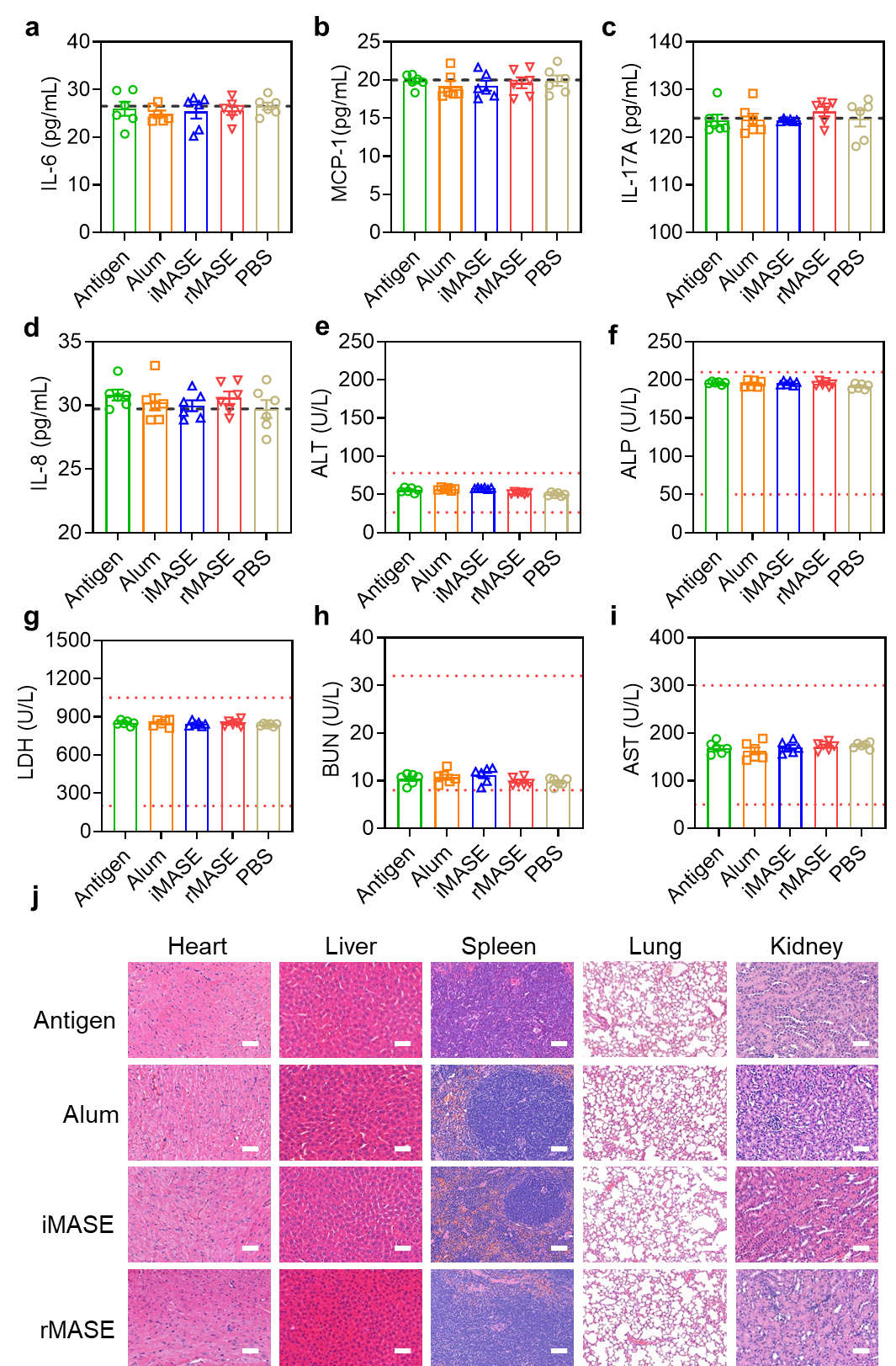


**Supplementary Fig. 13. The safety profile of rMASE.**

**(a)** IL-6, **(b)** MCP-1, **(c)** IL-17A, and **(d)** IL-8 levels in serum on day 28. The dotted line indicated the average concentrations of the untreated group (PBS). Healthy mice were intramuscularly administered with the indicated formulations, and serum was collected on day 28 for analysis. Analysis of **(e)** alanine aminotransferase (ALT), **(f)** alkaline phosphatase (ALP), **(g)** lactate dehydrogenase (LDH), **(h)** blood urea nitrogen (BUN), and **(i)** aspartate aminotransferase (AST). Healthy mice were intramuscularly administered with the indicated formulations, and serum was collected on day 28 for biochemical analysis. The value between the two lines represented the normal level. **(j)** Histopathological evaluations of the vital organs from the BALB/c mice. Healthy mice (n = 6) were intramuscularly administered with the indicated formulations, 28 days after administration, the heart, liver, spleen, lung, and kidney were harvested to stain with H&E. Scale bar: 100 μm. All data in the graphs were presented as arithmetic means ± s.e.m. from three independent experiments. For statistical analysis, a one-way analysis of variance was carried out with Tukey’s correction for multiple comparisons. **P* < 0.05, ***P* < 0.01, ****P* < 0.001.

The results showed that injection of the biomimetic vaccine caused no detectable abnormality in the above-mentioned biochemical parameters, verifying excellent biocompatibility.


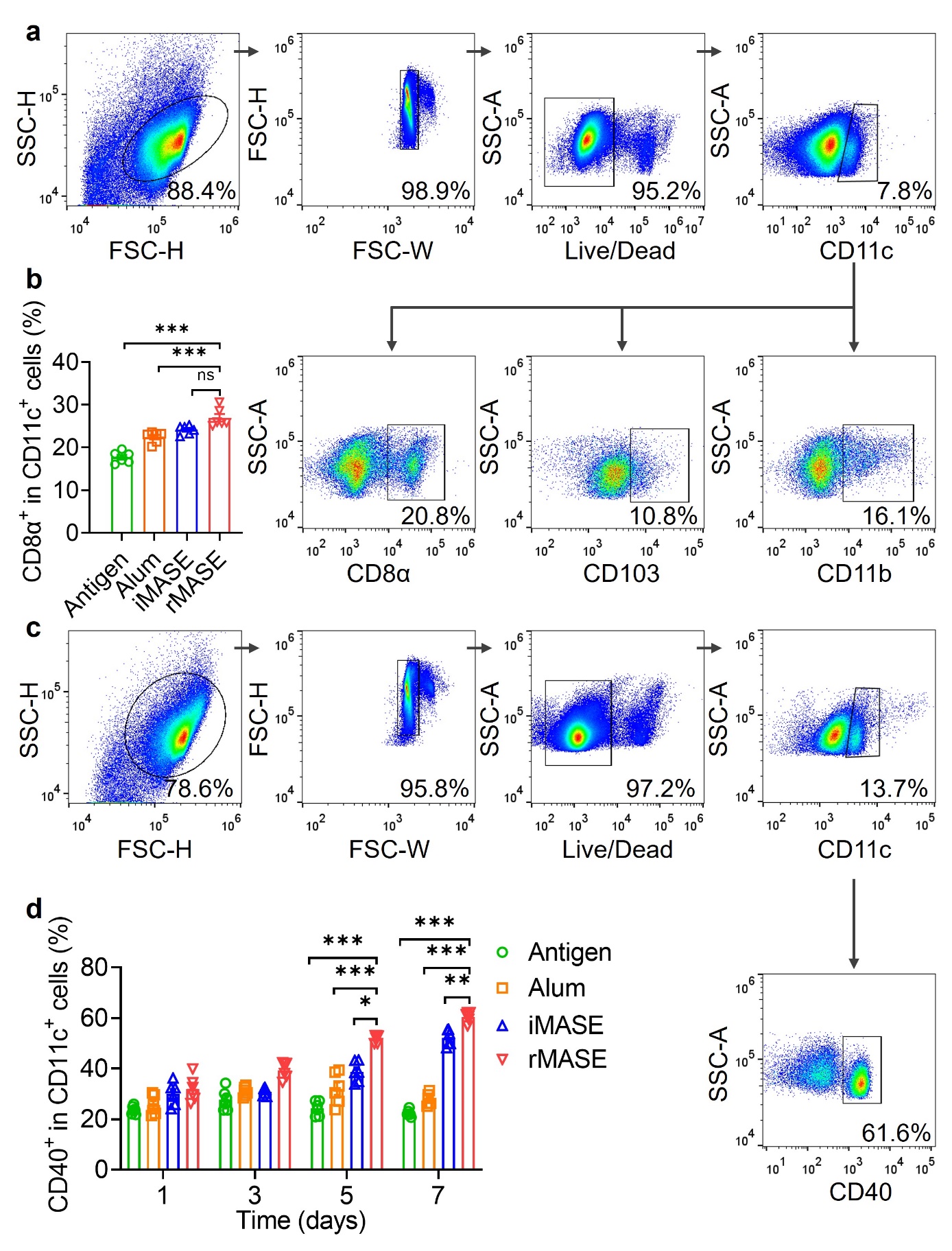


**Supplementary Fig. 14. DC subsets and activation in lymph nodes.**

**(a)** Gate strategy and **(b)** frequencies of DC subsets in lymph nodes (LNs). **(c)** Gate strategy and **(d)** population of CD40-expressing DCs in LNs. All data were presented as arithmetic means ± s.e.m. from three independent experiments. One-way or two-way analysis of variance was carried out with Tukey’s correction for multiple comparisons. **P* < 0.05, ***P* < 0.01, ****P* < 0.001.


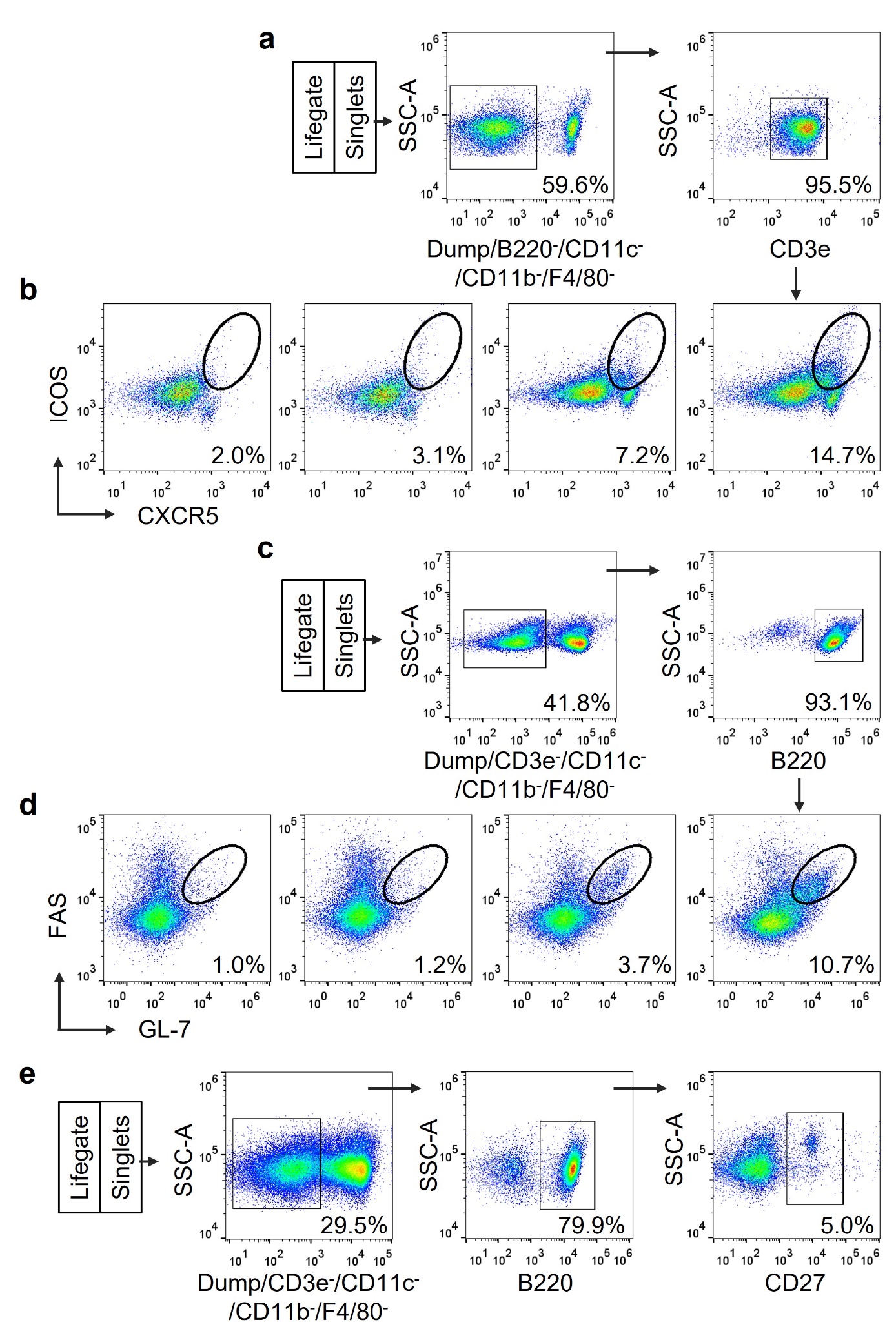


**Supplementary Fig. 15. Representative flow cytometry analysis of the germinal center and memory response.**

**(a)** Gate strategy and **(b)** population of ICOS^+^ CXCR5^+^ CD3^+^ T cell. **(c)** Gate strategy and **(d)** population of FAS^+^ GL-7^+^ B220^+^ cells. **(e)** Gate strategy of CD27^+^ B220^+^ cells.


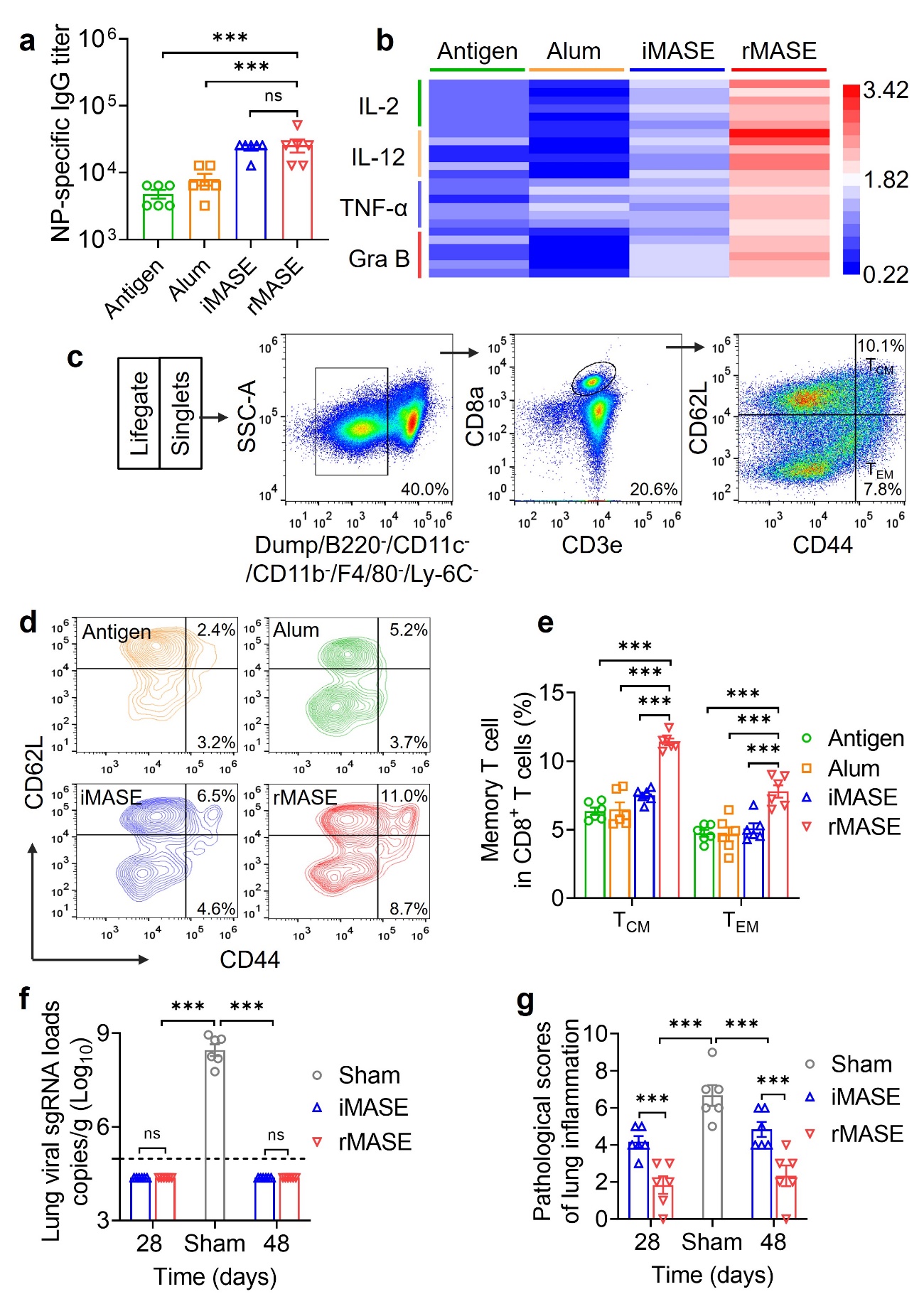


**Supplementary Fig. 16. rMASE induced potent immune response against SARS-CoV-2.**

**(a)** NP-specific IgG titer. Healthy mice were intramuscularly administered with the indicated formulations, and serums were collected on day 28 for antigen-specific antibody analysis. **(b)** Heatmap of the cytokines array. Comparison of the secretion profile of cytokines (IL-2, IL-12, TNF-α, and granzyme B) in the supernatant of the *ex vivo* stimulated splenocytes. ELISA analysis of cytokines from the splenocytes after *ex vivo* restimulation with RBD on day 28. The red color indicated the upregulation of the cytokines. **(c)** Gate strategy and **(d)** Representative data of the effector memory T cells (CD44^high^ CD62L^low^) and central memory T cells (CD44^high^ CD62L^high^) among CD3^+^ CD8^+^ splenocytes. **(e)** Frequency of T_CM_ and T_EM_ in CD8^+^ T cells on day 28. T_CM_ and T_EM_ were identified by CD3^+^ CD8^+^ CD44^high^ CD62L^high^ and CD3^+^ CD8^+^ CD44^high^ CD62L^low^, respectively. **(f)** SARS-CoV-2 titration from lung tissues. SARS-CoV-2 titration from lung tissues was evaluated by probing virus subgenomic RNA (sgRNA) using RT-qPCR. **(g)** Histopathology scores of overall lung lesions and pulmonary alveolar congestion. All data in the graphs were presented as arithmetic means ± s.e.m. from three independent experiments. For statistical analysis, a one-way analysis of variance was carried out with Tukey’s correction for multiple comparisons. **P* < 0.05, ***P* < 0.01, ****P* < 0.001.


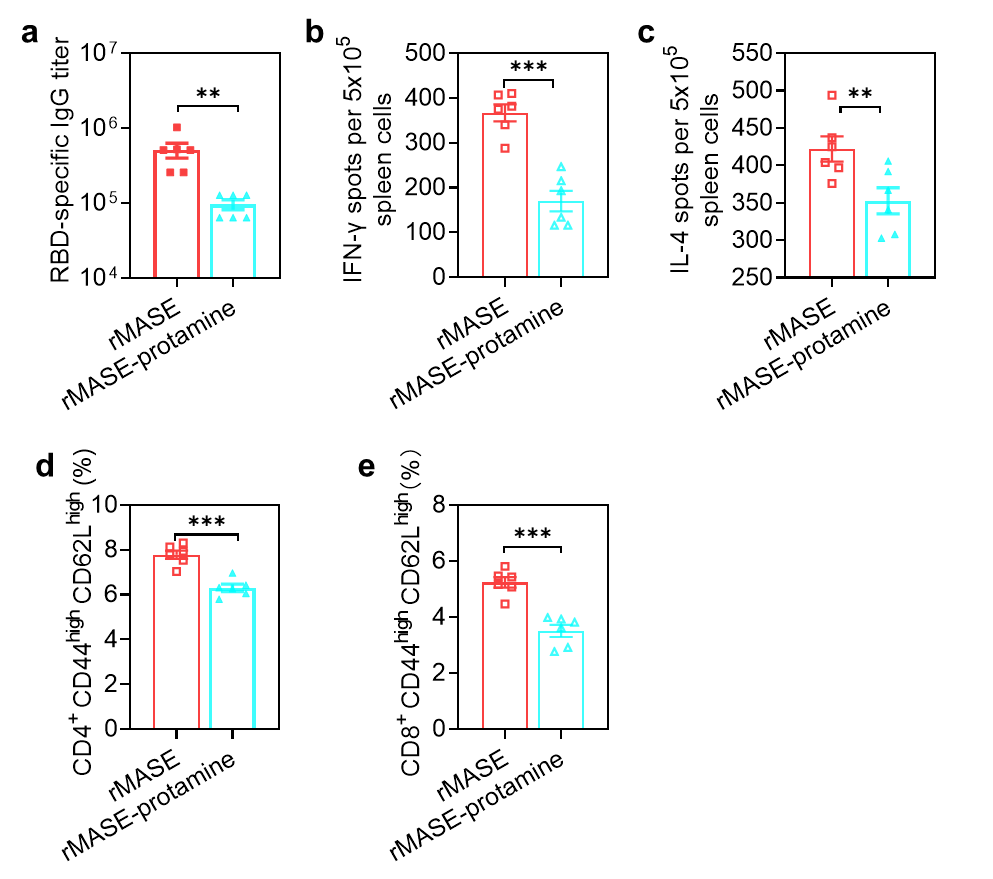


**Supplementary Fig. 17. Evaluation of the charge effect on inside-out strategy via the comparison with the protamine-delivered formulation.**

**(a)** Serum RBD-specific IgG titer. ELISPOT analysis of **(b)** IFN-γ and **(c)** IL-4 spot-forming cells among splenocytes. Flow cytometry of central memory T cells (CD44^high^ CD62L^high^) among **(d)** CD4^+^ T cells and **(e)** CD8^+^ T cells. All data in the graphs were presented as arithmetic means ± s.e.m. from three independent experiments. Unpaired Student’s t was carried out for statistical analysis. **P* < 0.05, ***P* < 0.01, ****P* < 0.001.

To screen the influence of the positive charges of NP on the enhanced immunogenicity of the inside-out strategy, we compared the immune results of rMASE with the co-delivery of RBD and protamine, a commonly employed cationic protein. To achieve this, RBD and protamine were consecutively loaded on the inside and outside of the multi-layered alum-stabilized emulsion (rMASE-protamine). Subsequently, rMASE-protamine and rMASE were administrated intramuscularly twice at a 14-day interval. As shown in Supplementary Fig. 17, rMASE-protamine failed to induce comparable RBD-specific antibody titer, IFN-γ-spot-forming cells, and the immune memory with rMASE, indicating the limited effect of antigen charge in the immunogenicity of the inside-out strategy.


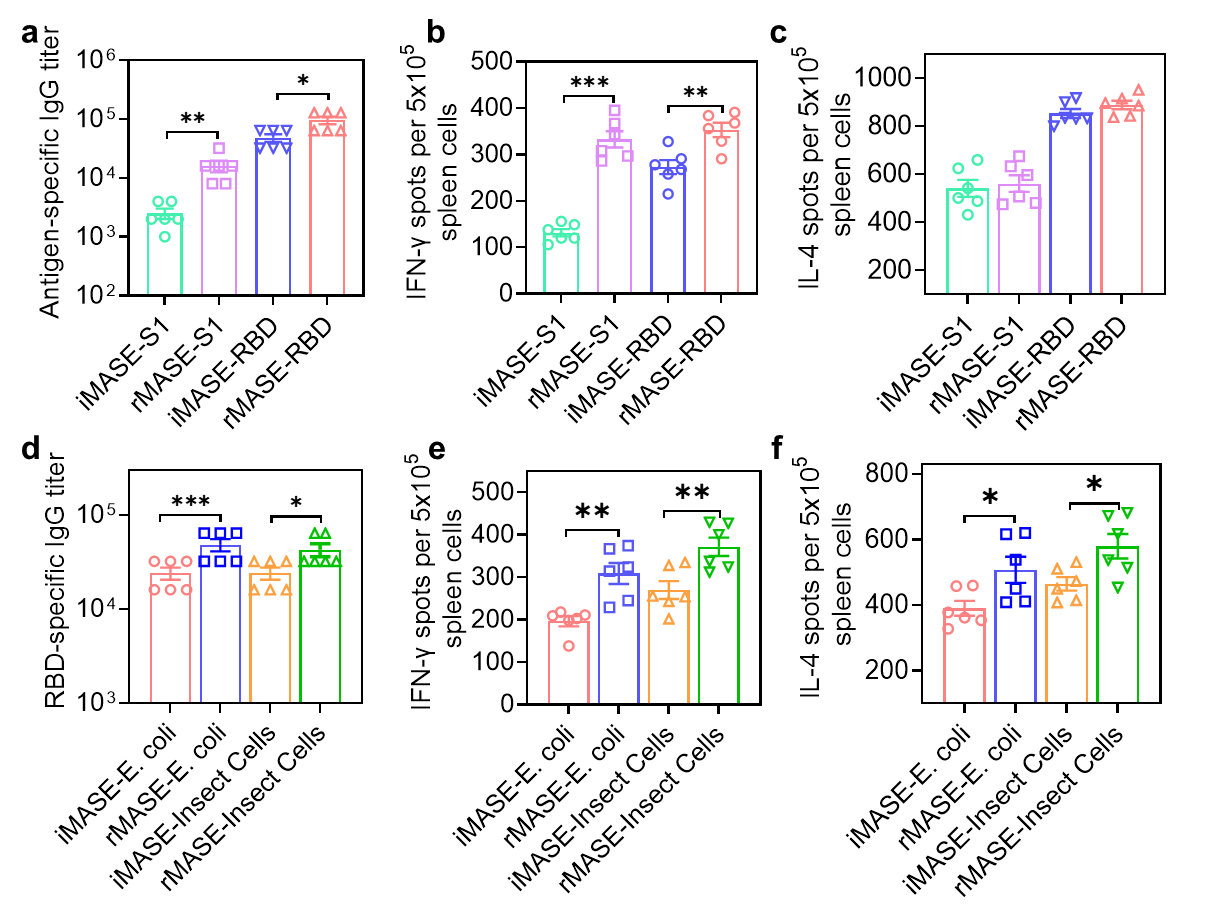


**Supplementary Fig. 18.** **rMASE assembled from different types of surface antigens and NPs for enhanced immune responses.**

**(a-c)** Both S1 protein- and monomer-RBD-formulated rMASE induced enhanced immune responses. **(a)** Serum antigen-specific IgG titer. ELISPOT analysis of **(b)** IFN-γ and **(c)** IL-4 spot-forming cells among splenocytes. **(d-f)** rMASE, combined with NP from both Escherichia coli (*E. coli*) and the eukaryotic cell lines, increased the immune responses. **(d)** Serum RBD-specific IgG titer. ELISPOT analysis of **(e)** IFN-γ and **(f)** IL-4 spot-forming cells among splenocytes. Mice were administrated intramuscularly with indicated formulations, and the immune responses were detected on day 28. All data in the graphs were presented as arithmetic means ± s.e.m. from three independent experiments. Unpaired Student’s t was carried out for statistical analysis. **P* < 0.05, ***P* < 0.01, ****P* < 0.001.

To evaluate whether the sequences of the antigens affect the immune response of the inside-out strategy, we constructed the RBD- or S1-formulated rMASE. As shown in the results, either S1 protein- or RBD-loaded rMASE, induced evidently increased antibody secretion and Th1-mediated responses, which indicated that the potentiated effect was not restrained by the sequence. Therefore, we speculated that the inside-out strategy may be applied to a broader range of antigens for the enhanced SARS-CoV-2 vaccination against the prevailing pandemics.

Moreover, to evaluate whether the antigen sources affect the immune response of rMASE, we constructed the formulation using NP from *E. coli* and the eukaryotic cell lines. From the results, we found that NPs from *E. coli* or the eukaryotic cell lines-loaded rMASE induced evidently increased antibody secretion and cellular immune responses, which indicated that the potentiated effect was not restrained by the antigen sources.


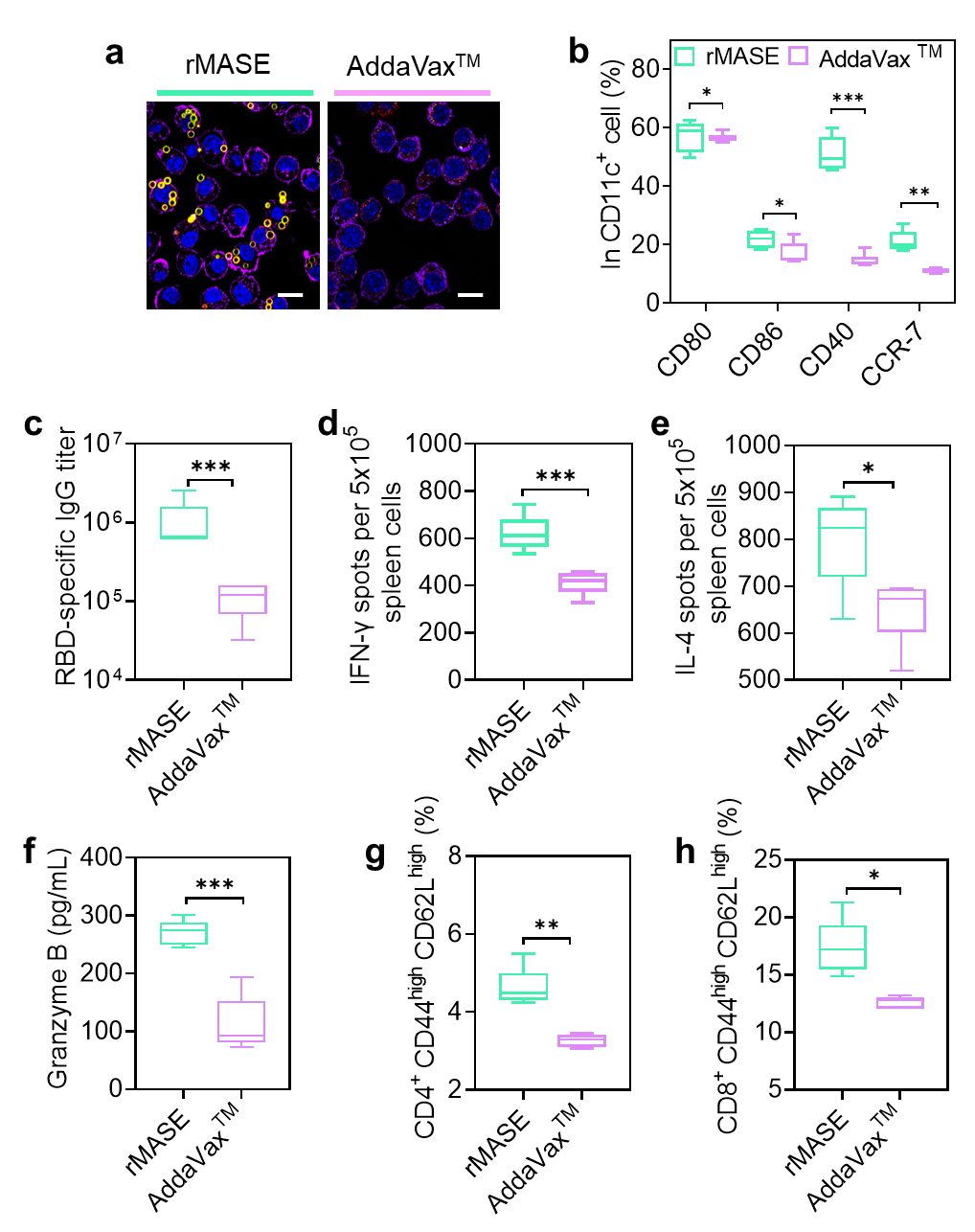


**Supplementary Fig. 19. A comparison of the immune response to rMASE and AddaVax^TM^.**

**(a)** Confocal images of the antigen uptake. DCs membrane, RBD, and NP were labeled with FITC-phalloidin (purple), Cy3 (red), and Cy5 (green), respectively. Scale bar: 10 μm. **(b)** DC activation after treating with the formulations *in vitro*. (**c**) Serum RBD-specific IgG titer. ELISPOT analysis of **(d)** IFN-γ and **(e)** IL-4 spot-forming cells among splenocytes. **(f)** The secretion of Granzyme B among the splenocytes. Flow cytometry of central memory T cells (CD44^high^ CD62L^high^) among **(g)** CD4^+^ T cells and **(h)** CD8^+^ T cells. All data in the graphs were presented as arithmetic means ± s.e.m. from three independent experiments. Unpaired Student’s t was carried out for statistical analysis. **P* < 0.05, ***P* < 0.01, ****P* < 0.001.

Here, we compared the immune response of rMASE with the commercial adjuvant (AddaVax™, a similar formulation to MF59^®^). Healthy mice were intramuscularly administered with rMASE and AddaVax™, and the immune responses were evaluated on day 28. As the fluidic mixture of RBD, NP, and the surfactant-stabilized emulsion, AddaVax™ can hardly dictate the delivery sequence or the cellular enrichment of NP and RBD within a single cell. As a result, we found that AddaVax™ failed to elicit comparable IgG titer and cellular immune responses to rMASE. The results further validated the dominant role of the inside-out strategy, that it is the delivery of NP before RBD stimulated the IFN-I-mediated innate immunity and subsequently activated the adaptive immune responses.

**
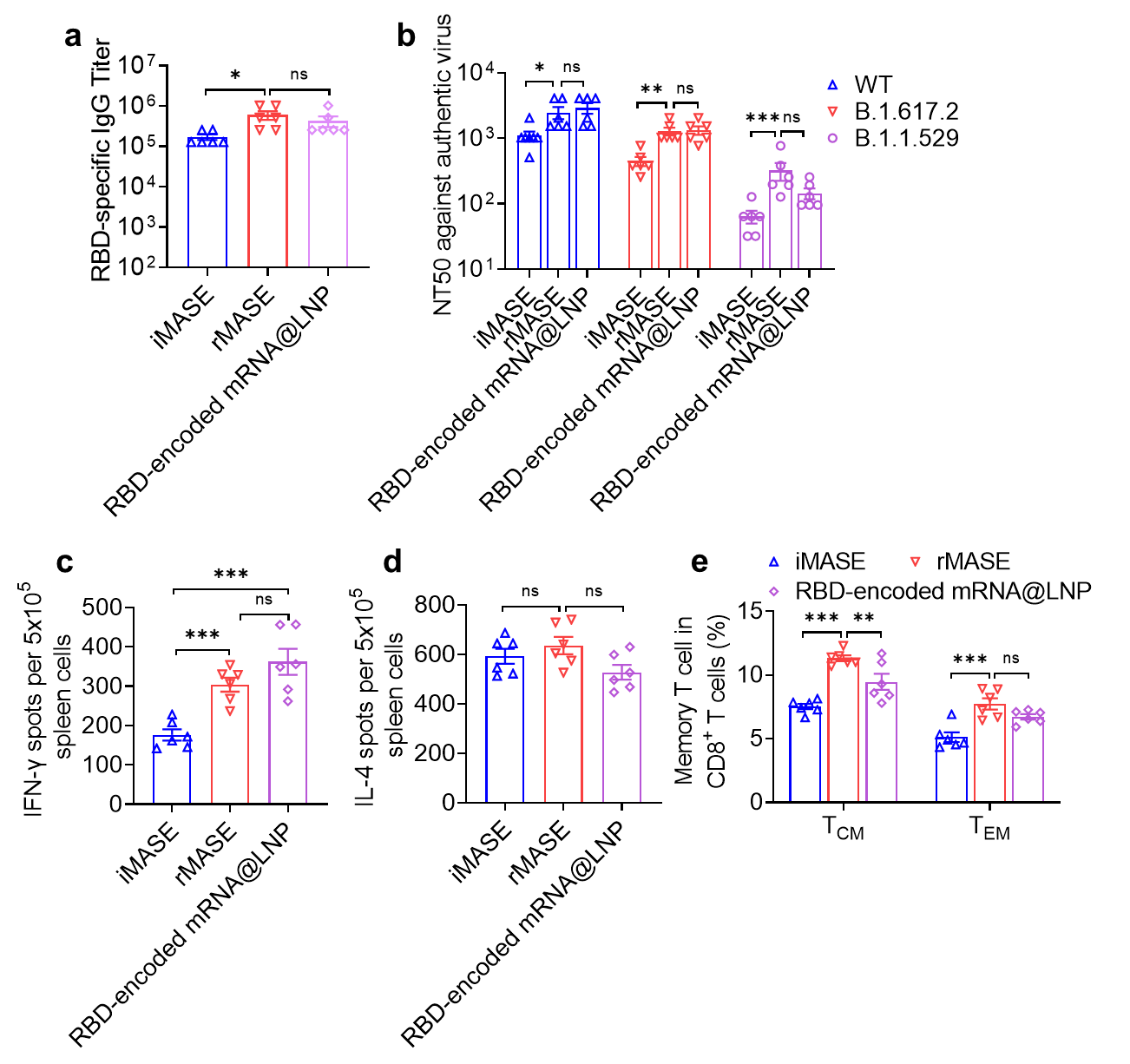
Supplementary Fig. 20. A comparison of RBD-encoded mRNA@LNP with rMASE in terms of immune effectiveness.**

**(a)** Serum RBD-specific IgG titer on day 28. **(b)** Serum neutralizing activity was evaluated by authentic SARS-CoV-2 mutants, illustrated by the serum half-maximal neutralizing titer (NT50) against live SARS-CoV-2 WT, B.1.617.2 and B.1.1.529. The number represented the fold decrease in neutralizing antibody titer. ELISPOT assay on **(c)** IFN-γ and **(d)** IL-4 spot-forming cells among the splenocytes, following stimulation with RBD. **(e)** Flow cytometry on the effector memory T cells (CD44^high^ CD62L^low^) and central memory T cells (CD44^high^ CD62L^high^) among CD3^+^ CD8^+^ splenocytes. All data in the graphs were presented as arithmetic means ± s.e.m. from three independent experiments. For statistical analysis, a one-way analysis of variance was carried out with Tukey’s correction for multiple comparisons. **P* < 0.05, ***P* < 0.01, ****P* < 0.001.


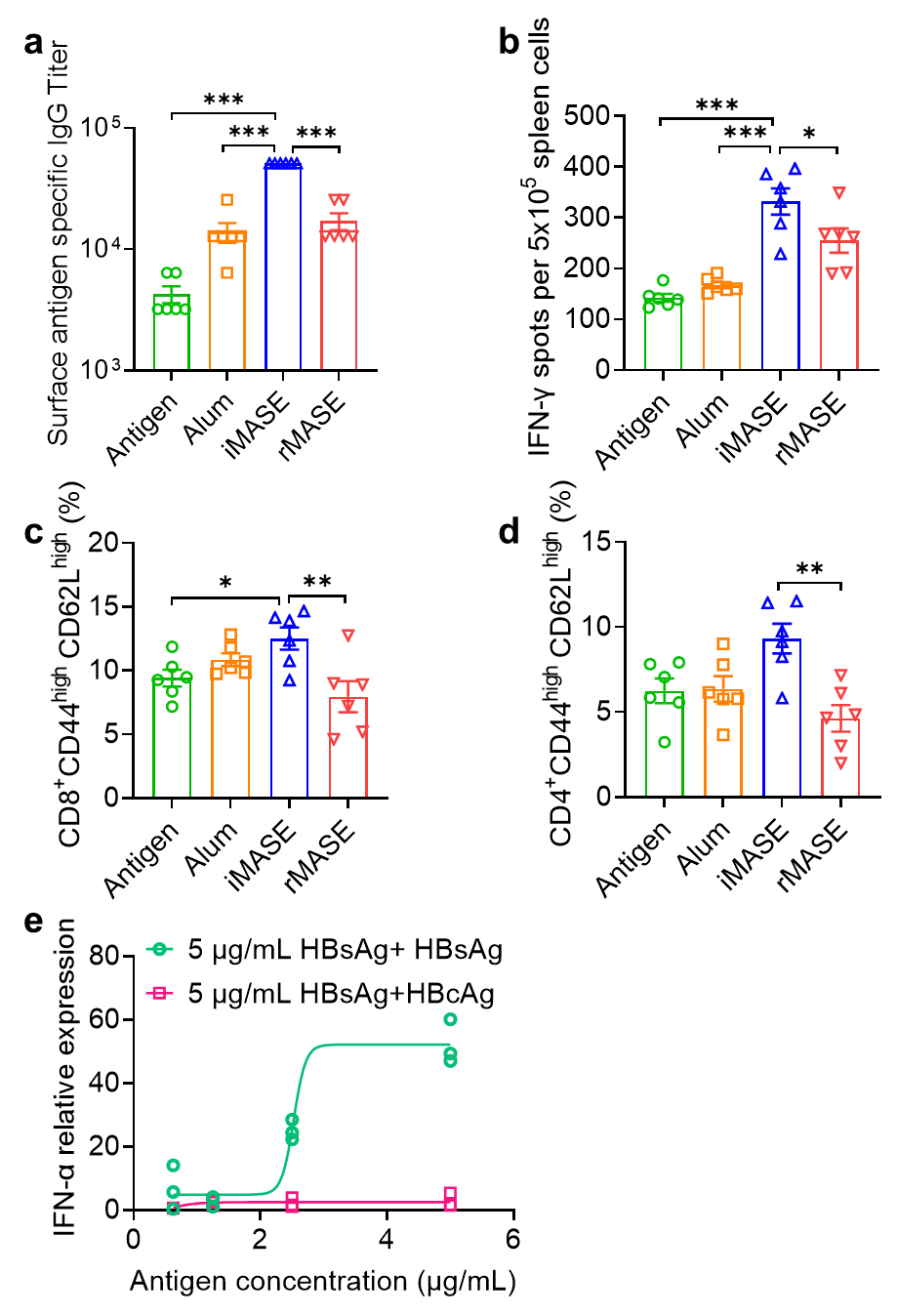


**Supplementary Fig. 21. The sequential delivery of the** **core and surface antigen** **of Hepatitis B virus via MASE system.**

**(a)** Serum HBsAg-specific IgG titer. **(b)** ELISPOT analysis of HBsAg-specific IFN-γ^+^ T cell. Flow cytometry of central memory T cells (CD44^high^ CD62L^high^) among **(c)** CD8^+^ T cells and **(d)** CD4^+^ T cells. **(e)** The expression of IFN-α among the BMDCs after treatment with the surface antigen (HBsAg) and core antigen (HBcAg) of HBV. For HBV, HBsAg and HBcAg were used to treat BMDCs, and the mRNA expression of IFN-α was determined using RT-qPCR. **(a-d)** These data in the graphs were presented as arithmetic means ± s.e.m. from three independent experiments. For statistical analysis, a one-way analysis of variance was carried out with Tukey’s correction for multiple comparisons. **P* < 0.05, ***P* < 0.01, ****P* < 0.001. **(e)** These data were analyzed by nonlinear-regression (dose-response-stimulation).

We have applied the inside-out strategy to HBV vaccinations. Through rMASE, we reversed the exposure sequence by loading the HBsAg (surface antigen) within the inner layer, and adsorbing the HBcAg (core antigen) on the surface. As shown in Supplementary Fig. 21a-d, for the iMASE, antigen-specific antibody secretion was increased by 3-fold, and IFN-γ T cells were boosted by 130% (*P* < 0.001), compared with rMASE. Furthermore, central memory T cells among CD4^+^ T cells and CD8^+^ T cells were also boosted. These data indicated that the inside-out strategy may not be applied to increase the efficacy of HBV vaccinations.

Furthermore, increasing the dose of the surface antigen boosted the IFN-α expression. However, the IFN-α levels were not increased with a higher dose of core antigen in the system, indicating that the inside-out strategy may not be applicable to the delivery of the antigens from the DNA-based viruses.

**Supplementary Table 1. H1N1 influenza antigen information**

HA and NP from A/PR/8/34/1934 (H1N1) were selected as the model antigens.^1,2^ The molecular weights of HA and NP were 59.0 kDa and 56.7 kDa, respectively.

An Easy-nano LC 1000 system coupled to a Q-Exactive mass spectrometer (Thermo Fisher Scientific, San Jose, CA) was used to analyze the impurities of NP. The sample concentration was measured by NANODROP ONE (Thermo Fisher Scientific, San Jose, CA) to control approximately the same loading amount of each sample. MaxQuant software was used to analyze MS raw files to identify proteins, version 1.6.3.3.

| Component | Percent (%) |
| --- | --- |
| Keratin, type II cytoskeletal 1 | 0.52 |
| Prohibitin 1 | 0.39 |
| Immunoglobulin lambda constant 2 | 0.32 |
| Keratin, type I cytoskeletal 9 | 0.31 |
| Albumin | 0.29 |
| Keratin, type I cytoskeletal 10 | 0.22 |
| Heterogeneous nuclear ribonucleoproteins A2/B1 | 0.21 |
| Tubulin beta chain | 0.20 |
| Heterogeneous nuclear ribonucleoprotein A1-like 3 | 0.20 |
| Lysozyme C | 0.20 |
| Dermcidin | 0.19 |
| 40S ribosomal protein S3a | 0.17 |
| Beta-2-microglobulin | 0.16 |
| 60S ribosomal protein L34 | 0.15 |
| Heat shock cognate 71 kDa protein | 0.13 |
| Polyubiquitin-C | 0.11 |
| Cleavage and polyadenylation specificity factor subunit 5 | 0.10 |
| 60 kDa heat shock protein, mitochondrial | 0.10 |
| Endoplasmic reticulum chaperone BiP | 0.09 |
| 40S ribosomal protein S2 | 0.09 |
| Zymogen granule protein 16 homolog B | 0.08 |
| Keratin, type II cytoskeletal 2 epidermal | 0.07 |
| Heat shock 70 kDa protein 1-like | 0.06 |
| Prohibitin-2 | 0.06 |
| Immunoglobulin heavy constant alpha 1 | 0.06 |
| ATPase family AAA domain-containing protein 3C | 0.06 |
| Poly [ADP-ribose] polymerase 1 | 0.05 |
| ATP-dependent RNA helicase A | 0.05 |
| Vimentin | 0.04 |
| RPA-related protein RADX | 0.03 |
| DNA topoisomerase 1 | 0.03 |
| Peroxisomal multifunctional enzyme type 2 | 0.03 |
| Ras GTPase-activating-like protein IQGAP1 | 0.02 |
| Deleted in malignant brain tumors 1 protein | 0.01 |

**Supplementary Table 2.** **Characterization of the multi-layered Pickering emulsions**

iMASE and rMASE were prepared according to the indicated method. The size distributions and zeta potential were determined by DLS via nano zeta sizer. The loading efficiency was evaluated using the equation: Antigen loading efficiency (%) = (total antigens (mg/mL) - fluidic antigens (mg/mL)) / total antigens (mg/mL) ×100%. Data were demonstrated as mean ± s.e.m. (n = 3).

| Group | | Size  (nm) | Zeta potential | | | Antigen loading efficiency (%) | |
| --- | --- | --- | --- | --- | --- | --- | --- |
|  |  |  | (mv) | HA | | | NP |
| iMASE | | 3504.33 ± 91.34 | 34.43 ± 0.35 | | 98.63 ± 0.21 98.61 ± 0.26 | | |
| rMASE | 3567.33 ± 184.43 | | 38.10 ± 0.62 | | 98.56 ± 0.30 98.50 ± 0.24 | | |

**Supplementary Table 3. Formulations of the treated groups for H1N1 influenza**

For the *in vivo* analysis, all formulations were administered with the same number of antigens, regardless of the different antigen loading efficiency. Formulations were intramuscularly injected in a volume of 100 uL per mouse for a total of two injections.

| Group | Alhydrogel^®^adujvant 0.1% (w/v) | Squalene  (5%, v/v) | HA | NP |
| --- | --- | --- | --- | --- |
| Antigen | 0 µg | 0 µL | 5 µg | 5 µg |
| Alum | 100 µg | 0 µL | 5 µg | 5 µg |
| iMASE | 100 µg | 5 µL | 5 µg | 5 µg |
| rMASE | 100 µg | 5 µL | 5 µg | 5 µg |

**Supplementary Table 4. The information of SARS-CoV-2 antigens**

Recombinant RBD-sc-dimer of spike protein was selected as the model antigen, which was designed by connecting two RBD (S protein residues 319–537). The NP was constructed according to the SARS-CoV-2 sequence published on NCBI.^3^

| Antigen | Accession number | Molecular weight (kDa) |
| --- | --- | --- |
| RBD | YP_009724390 | 61.7 |
| NP | YP_009724397 | 48.0 |

**Supplementary Table 5. Formulation of the treated groups for SARS-CoV-2**

For the *in vivo* analysis, all formulations were administered with the same number of antigens, regardless of the different antigen loading efficiency. Formulations were intramuscularly injected in a volume of 100 uL per mouse every two weeks for a total of two injections.

| Group | Alhydrogel^®^adujvant 0.1% (w/v) | Squalene  (5%, v/v) | RBD | NP |
| --- | --- | --- | --- | --- |
| Antigen | 0 µg | 0 µL | 5 µg | 5 µg |
| Alum | 100 µg | 0 µL | 5 µg | 5 µg |
| iMASE | 100 µg | 5 µL | 5 µg | 5 µg |
| rMASE | 100 µg | 5 µL | 5 µg | 5 µg |

**Supplementary Table 6.** **Kinetic parameters for binding of iMASE and rMASE to the RBD antigen by biolayer interferometry**

| Analyte | KD (M) | KD Error | kon (1/Ms) | kon Error | kdis (1/s) | kdis Error |
| --- | --- | --- | --- | --- | --- | --- |
| iMASE | 2.24× 10^-8^ | 4.71× 10^-9^ | 1.06× 10^4^ | 1.99× 10^3^ | 2.38× 10^-4^ | 2.26× 10^-5^ |
| rMASE | 2.58× 10^-10^ | 2.89× 10^-11^ | 3.63× 10^4^ | 1.00× 10^3^ | 9.38× 10^-6^ | 1.05× 10^-6^ |

**Reference**

1 Schoch, C. L. *et al.* NCBI Taxonomy: a comprehensive update on curation, resources and tools. *Database (Oxford)*, (2020).

2 Winter, G. & Fields, S. The structure of the gene encoding the nucleoprotein of human influenza virus A/PR/8/34. *Virology* **114**, 423-428, (1981).

3 Wu, F. *et al.* A new coronavirus associated with human respiratory disease in China. *Nature* **579**, 265-269, (2020).
